# Protective Effect of Remdesivir Against Pulmonary Fibrosis in Mice

**DOI:** 10.3389/fphar.2021.692346

**Published:** 2021-08-26

**Authors:** Xiaohe Li, Rui Liu, Yunyao Cui, Jingjing Liang, Zhun Bi, Shimeng Li, Yang Miao, Liang Zhang, Xiaoping Li, Honggang Zhou, Cheng Yang

**Affiliations:** ^1^ State Key Laboratory of Medicinal Chemical Biology, College of Pharmacy and Tianjin Key Laboratory of Molecular Drug Research, Nankai University, Tianjin, China; ^2^ Tianjin Key Laboratory of Molecular Drug Research, Tianjin International Joint Academy of Biomedicine, Tianjin, China; ^3^ Department of Thoracic Surgery, Tian Jin First Central Hospital, Tianjin, China

**Keywords:** remdesivir (RDV), COVID-19, pulmonary fibrosis, bleomycin, TGF-β1

## Abstract

Pulmonary fibrosis is a known sequela of severe or persistent lung damage. Existing clinical, imaging and autopsy studies have shown that the lungs exhibit a pathological pulmonary fibrosis phenotype after infection with coronaviruses, including severe acute respiratory syndrome coronavirus 2 (SARS-CoV-2). Pulmonary fibrosis may be one of the most serious sequelae associated with coronavirus disease 2019 (COVID-19). In this study, we aimed to examine the preventative effects of the antiviral drug remdesivir on pulmonary fibrosis. We used a mouse model of bleomycin-induced pulmonary fibrosis to evaluate the effects of remdesivir on pulmonary fibrosis *in vivo* and further explored the potential pharmacological mechanisms of remdesivir in lung fibroblasts and alveolar epithelial cells *in vitro*. The preventive remdesivir treatment was started on the day of bleomycin installation, and the results showed that remdesivir significantly alleviated bleomycin-induced collagen deposition and improved pulmonary function. *In vitro* experiments showed that remdesivir dose-dependently suppressed TGF-β1-induced lung fibroblast activation and improved TGF-β1-induced alveolar epithelial to mesenchymal transition. Our results indicate that remdesivir can preventatively alleviate the severity of pulmonary fibrosis and provide some reference for the prevention of pulmonary fibrosis in patients with COVID-19.

## Introduction

The novel coronavirus severe acute respiratory syndrome coronavirus 2 (SARS-CoV-2) causes coronavirus disease 2019 (COVID-19), was identified in December 2019 and has triggered an outbreak and spread rapidly across the world (Wang et al., 2020). As of January 7, 2021, SARS-COV-2 is still prevalent, with a total of 87.7 million confirmed cases worldwide (WHO, 2020), but there are still no effective treatments. SARS-CoV-2 is the sixth international public health emergency after the emergence of H1N1, poliovirus, Ebola virus, and Zika virus (Lai et al., 2020). The SARS-CoV-2 pandemic not only brings physical and psychological burdens to patients but also had a major impact on the development of all aspects of society.

Most COVID-19 patients have common respiratory and gastrointestinal symptoms, such as fever, dry cough and diarrhea, and some patients may progress to severe manifestations, including acute respiratory distress syndrome (ARDS), respiratory failure, multiorgan failure, and even death (Bajwah et al., 2020; Wang et al., 2020). According to former reports, many patients survive the acute phase of ARDS but die as a result of subsequent progressive pulmonary fibrosis (Meduri et al., 1995). A large amount of clinical evidence also confirms that coronavirus infection in the respiratory system can cause lung damage and develop into irreversible pulmonary fibrosis (George et al., 2020). For example, Xie et al. showed that 45% of patients infected with SARS-CoV develop the appearance of “ground glass” (a sign of fibrosis) in the lungs (Xie et al., 2005). Up to 33% of patients infected with Middle East respiratory syndrome (MERS)-CoV have pulmonary fibrosis sequelae (Das et al., 2017). SARS-CoV-2 has similar gene sequences as SARS-CoV and MERS-CoV, with consistencies of 79 and 50%, respectively (Jiang et al., 2020; Lu et al., 2020). Therefore, pulmonary fibrosis may also be the main sequelae in SARS-CoV-2 patients during the recovery period. Recently, a clinical study showed the formation of fibrosis during the rehabilitation of patients with COVID-19 (Xu et al., 2020). Other studies also showed that 17.5–33.9% of COVID-19 patients had fibrotic changes on chest CT scans (Pan et al., 2020; Zhou et al., 2020). Hence, although long-term follow-up studies are needed to determine the exact proportion of post-COVID-19 fibrosis, antifibrotic therapy should be a key concern for COVID-19 survivors.

Remdesivir is a nucleoside analog drug that inhibits viral RNA-dependent RNA polymerase (RdRP) through triphosphate metabolites, which can stop the transcription of viral RNA and prevent viral replication (Warren et al., 2016; Tchesnokov et al., 2019). In 2015, the FDA approved remdesivir for the treatment of Ebola virus (Mulangu et al., 2019); while remdesivir did not show significant efficacy in the treatment of Ebola virus but did show significant antiviral activity against coronaviruses such as SARS-CoV and MERS-CoV (Brown et al., 2019; de Wit et al., 2020). Recently, a series of *in vitro* and *in vivo* studies showed that remdesivir also has a strong inhibitory effect on SARS-CoV-2 (Sheahan et al., 2020). Some clinical trials and case reports also support remdesivir as a potentially effective drug for the treatment of COVID-19 (Beigel et al., 2020; Goldman et al., 2020; Spinner et al., 2020). At present, remdesivir has been approved by the FDA and EMA for the treatment of COVID-19 (Agency, 2020; Diseases, 2020). However, the effect of remdesivir on subsequently occurring pulmonary fibrosis in patients with COVID-19 has not been reported.

Since our laboratory is not qualified for viral testing, we used a bleomycin (BLM)-induced pulmonary fibrosis mouse model to evaluate the preventative effect of remdesivir on pulmonary fibrosis and further explored the potential pharmacological mechanisms, which may provide a reference for the clinical prevention of pulmonary fibrosis in COVID-19 patients.

## Materials and Methods

### Materials

Recombinant human transforming growth factor-β1 (TGF-β1) was purchased from Peprotech (United States). Antibodies against collagen Ⅰ, fibronectin, P-Smad3/Smad3, P-Akt/Akt, P-ERK/ERK, P-JNK/JNK, P-P38/P38, GAPDH, E-cadherin, vimentin, and N-cadherin were purchased from Cell Signaling Technology (United States). The β-tubulin antibody was obtained from Proteintech (China). The α-SMA antibody was obtained from Santa Cruz Biotechnology (China). Goat pAbs against Rb IgG (HRP) and Rb pAbs against Ms IgG were obtained from ImmunoWay (China). TRIzol reagent was acquired from Ambion Life Technology (China). DEPC-treated H_2_O and RNase Away H_2_O were purchased from Life Technologies. Fastking gDNA Dispelling RT SuperMix was purchased from Tiangen (Beijing, China). UNICON® qPCR SYBR Green Master Mix and liposomal transfection reagents were purchased from Yevsen (Shanghai, China). The Masson’s trichrome staining kit was obtained from Solarbio (China). Recombinant Mouse Platelet-derived Growth Factor-BB were purchased from MedChemExpress (China).

### Cell Culture

Human pulmonary epithelial cells (A549, ATCC) were maintained in RPMI-1640 (KeyGEN BioTECH, China) with 10% fetal bovine serum (FBS, ExCell Bio, China). Mouse embryonic fibroblasts (NIH-3T3, ATCC) were maintained in DMEM (KeyGEN BioTECH, China) with 10% FBS. NIH-3T3 cells were stably transfected with the (CAGA)_12_-Lux reporter to generate CAGA-NIH-3T3 reporter cells. All cells were incubated with 5% CO_2_ at 37°C.

### Animals

Male C57BL/6 mice (6–8 weeks old, 20–25 g) were purchased from the Weitong Lihua Experimental Animal Technology Co. Ltd. (Beijing, China). The animal experimental protocol was performed in accordance with the Health Guide for Care from the National Institutes (NIH Publications No. 85–23, revised 1996). All animal care and experimental procedures complied with guidelines approved by the Institutional Animal Care and Use Committee (IACUC) of Nankai University (Permit No. SYXK 2019-0001).

### BLM Administration

A mouse pulmonary fibrosis model was established by intratracheal BLM administration as described previously (Guo et al., 2019). Briefly, mice were intratracheally administered BLM (2 U/ Kg) dissolved in physiological saline (0.9% NaCl). Forty mice were randomly divided into five groups: the NaCl group, the BLM group, the positive control group (BLM + nintedanib, 100 mg kg^−1^), the low-dose remdesivir group (BLM + remdesivir, 10 mg kg^−1^) and the high-dose remdesivir group (BLM + remdesivir, 20 mg kg^−1^). The mice in the remdesivir groups were intraperitoneally injected daily with 10 mg kg^−1^ or 20 mg kg^−1^ remdesivir, which was suspended in 5% DMSO, 10% PEG and 2.5% polyoxyl 40-hydrogenated castor oil, while mice in the control and BLM groups received equal volumes of vehicle. Mice in the positive control group were intragastrically administered 100 mg kg^−1^ nintedanib daily. The mice were sacrificed on the 14th day after BLM administration for subsequent analysis, including hydroxyproline content determination.

### Isolation of Primary Pulmonary Fibroblasts

Primary pulmonary fibroblasts (PPF) were isolated from C57BL/6 J mice. Briefly, the lungs were lavaged three times with 1 ml of PBS, digested with 2.5 mg/ ml dispase II (Roche, United States), and 2.5 mg/ ml Collagenase Type 4 (Worthington, United States) for 30 min at 37°C and then centrifuged. The cell pellet was resuspended in DMEM containing 10% FBS and cultured in 5% CO_2_ at 37°C in a humidified atmosphere. PPF cells at passages two to five were used for various assays.

### Hydroxyproline Assay

Hydroxyproline was analyzed according to previously described methods (Li et al., 2019).

### Histological Examination

The left lung was fixed in 10% formalin, dehydrated and embedded in paraffin. Tissue sections with a thickness of 4 μm were incubated for 4 h at 60°C and stained with hematoxylin and eosin (H and E) and Masson’s trichrome. Quantification of pulmonary fibrosis was performed as described previously (Jiang et al., 2004). In brief, Masson staining slides were examined under low power and the whole lung images were captured. Images were analyzed by Image-Pro Plus version 6.0 to demarcate the entire lung area and automatically calculate the total pixel Pw of the region and then calculate the total pixel Pf of the fibrotic region (fibrosis ratio = fibrotic area total pixel Pf/total lung total pixel Pw).

### Pulmonary Function Testing

After the mice were anesthetized, the trachea was exposed, and a tracheal cannula was placed and fixed. The mice were transferred to a plethysmography chamber for pulmonary function analysis using an Anires2005 system according to the manufacturer’s instructions (Beijing Biolab, Beijing, China).

### Cell Viability Analysis

Cell viability was measured using 3-(4,5-dimethylthiazol-2-yl)-2,5-diphenyltetrazolium bromide (MTT) as previously described (Li et al., 2019).

### Luciferase Assay

Luciferase reporter assays were performed as previously described (Guo et al., 2019).

### Wound Healing Assay

Wound healing assays were performed as previously described (Li et al., 2019).

### Transwell Migration Assays

The influence of Remdesivir to NIH-3T3 cells migration was evaluated with a Transwell system. (Corning Costar, 3,422, United States) Briefly, serum-starved cells were trypsin-harvested in DMEM with 0.1% FBS. Next, 600 μL of medium containing 20% FBS was added to the lower chambers, while 200 μL NIH-3T3 cells (1 × 10^5^) were plated in the upper chambers. At the same time, NIH-3T3 cells were treated with/without PDGF-bb (10 ng/ ml) and remdesivir (12.5, 25, and 50 μM). After 24 h incubation, the cells on the bottom of the transwell membrane were fixed with 4% paraformaldehyde at 37 °C for 20 min and stained with 1% crystal violet at 37°C for 15 min, the nonmigrating cells in the upper chamber were removed with blunt-end swabs. The membranes were washed three times with PBS and photographed under a microscope.

### Quantitative Real-Time PCR

The mRNA expression level of genes was determined by qRT-PCR using primers according to a previously described protocol (Li et al., 2020). Primer pairs of target genes used were as follows:

Mouse β-actin (NM_007393.3), 5′-AGG​CCA​ACC​GTG​AAA​AGA​TG-3′ and 5′-AGAGCA TAG​CCC​TCG​TAG​ATG​G-3′; Human GAPDH (NM_001256799), 5′-GGA​GCG​AGA​TCC​CTC​CAA​AAT-3′ and 5′-GGC​TGT​TGT​CAT​ACT​TCT​CAT​GG-3′; Mouse α-SMA (NM_001297715.1), 5′-TGGGTGAACTCCATCGCTGTA-3′and 5′-GTCGAA TGCAACAAGGAAGCC-3′; Mouse Collagen Ⅰ (NM_000088.4), 5′-AAGCCGGAGGACAACCTTTTA-3′and 5′-GCG​AAG​AGA​ATG​ACC​AGA​TCC-3′; Mouse Fibronectin (NM_001306132.1), 5′-GTGCCCGGAATACGCATGTA-3′and 5′-CTG​GTG​GAC​GGG​ATC​ATC​CT-3′; Human E-cadherin (NM_0,01317185.2), 5′-A TTT​TTC​CCT​CGA​CAC​CCG​A T-3′and 5′- TCC​CAG​GCG​TAG​ACC​AAG​A-3′; Human Vimentin (NM_003380.5), 5′-AGT​CCA​CTG​AGT​ACC​GGA​GAC-3′ and 5′-CA TTTCACGCA TCTGGCGTTC-3′; Human N-cadherin (NM_001792.5), 5′-TTTGA TGG​AGG​TCT​CCT​AAC​ACC-3′ and 5′- ACG​TTT​AAC​ACG​TTG​GAA​A TGTG-3′; Mouse P16 (NM_058197), 5′-GGC​AGG​TTC​TTG​GTC​ACT​GT-3′ and 5′-TGT​TCA​CGA​AAG​CCA​GAG​CG-3′; Mouse P21 (NM_001111099), 5′-CCTGGTGATGTCCGACCTG-3′ and 5′-CCATGAGCGCATCGCAATC-3′.

### Western Blot Analysis

All proteins were collected from cells or lung tissues as reported previously (Ning et al., 2004). The monoclonal antibodies used in this study were: α-SMA (Affinity, BF9212), Collagen Ⅰ (Affinity, AF7001), Fibronectin (Affinity, AF5335), β-tubulin (Affinity, T0023), GAPDH (Affinity, AF7021), Phospho-Smad3 (Affinity, AF3362), Smad3 (Affinity AF6323), Smad2 (Affinity, AF6449), Phospho-Smad2 (Affinity, AF3450 Phospho-p38 (Affinity, AF4001), p38 (Affinity, BF8015), Phospho-JNK (Affinity, AF3318), JNK (Affinity, AF6318), Phospho-ERK (Affinity, AF1015), ERK (Affinity, AF0155), Phospho-AKT (Affinity, AF0832), AKT (Affinity, AF6212), Cleaved PARP (Affinity, AF7023), E-cadherin (Cell Signaling Technology, 14472S), N-cadherin (Cell Signaling Technology, 13,116), Vimentin (Cell Signaling Technology, 5,741). The monoclonal antibodies are diluted 1:1,000 with skimmed milk powder.

### Immunofluorescence Analysis

NIH-3T3 or A549 cells were seeded in a 24-well chamber. At the end of the treatment, the cells were fixed with a 4% fixative solution for 15 min (Solarbio), permeabilized with 0.2% Triton X-100 for 20 min, and incubated in 5% BSA for 30 min. NIH-3T3 cells were cultured overnight with α-SMA primary antibodies (Affinity, BF9212), and A549 cells were probed with E-cadherin (Cell Signaling Technology, 14472S) and vimentin antibodies (Cell Signaling Technology, 5,741) diluted in 5% BSA, the monoclonal antibodies are diluted 1:200 with skimmed milk powder. Then, the cells were incubated with TRITC-conjugated or FITC-conjugated secondary antibodies. The cells were washed with PBST, and DAPI (Beyotime Biotechnology) was used to stain the nuclei. The fluorescence was examined with a confocal microscope (Nikon, Japan).

### Immunohistochemical Staining

Paraffin-embedded lung tissue was dewaxed with xylene, and the sections were heated in a microwave oven with antigen-fixing solution (0.01 M citrate buffer) for 20 min. After the sections were cooled to room temperature and blocked with an immunohistochemistry kit, the primary antibody was added and incubated at 4 °C overnight. The primary antibodies were as follows: mouse anti-α-SMA (1:200 dilution, Affinity, BF9212), mouse anti-fibronectin (1:200 dilution, Affinity, AF5335). After being washed with TBST three times, the tissue sections were incubated with the secondary antibody at room temperature for 1 h. Subsequently, the tissue sections were rehydrated through a series of ethanol solutions and were stained with hematoxylin to observe histological changes and target gene expression under an optical microscope.

### Cell Cycle

NIH-3T3 cells were seeded in 6-well plates at a density of 1 × 10^6^cells/well and cultured at 37°C in a humidified 5% CO_2_ atmosphere until the cell confluency reached 60–70%. After treated with remdesivir at the desired concentrations (12.5, 25, and 50 μM), NIH-3T3 cells were washed with precooled phosphate buffered saline (PBS) twice and then fixed in 70% ethanol overnight at 4°C. The fixed cells which were washed twice with pre-cooled PBS after fixation were then stained with Propidium (PI)/Rnase A and incubated at 37°C in the dark for 30 min. The DNA content of NIH-3T3 cells were assessed by using a flow cytometer (Thermo Fisher Scientific). FlowJo software (Version 7.6.1) was used to analyze the data.

### Statistical Analysis

The data are presented as the means ± SD and were analyzed using Prism version 7.0 software. Differences between the experimental and control groups were assessed by Student’s t tests. Significant differences among multiple groups were analyzed by one-way ANOVA. A value of *p* < 0.05 was considered statistically significant.

## Results

### Remdesivir Attenuates BLM-Induced Pulmonary Fibrosis in Mice

**FIGURE 1:**
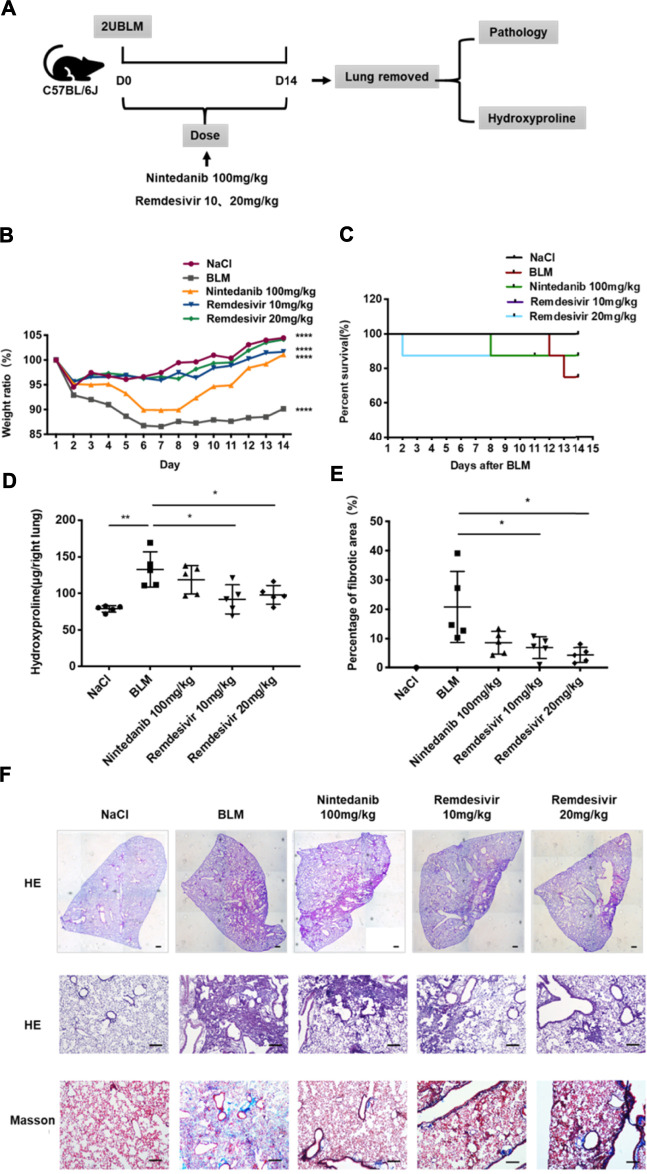
Remdesivir ameliorates BLM-induced pulmonary fibrosis in mice. **(A)** Dosing regimen in BLM-induced pulmonary fibrosis model. **(B)** Changes in body weight relative to initial body weight during administration. **(C)** Percentages of surviving mice were plotted from day 1–14 after BLM treatment. **(D)** Hydroxyproline contents of lung tissues in mice. **(E)** Statistics of lung fibrosis area among groups. **(F)** Lung tissue sections were stained with hematoxylin-eosin (H and E) and Masson Trichrome staining. Scale bar = 50 μm. Data was noted as the means ± SD, *n* = 5. * *p* < 0.05, ** *p* < 0.01, **** *p* < 0.0001.

**FIGURE 2:**
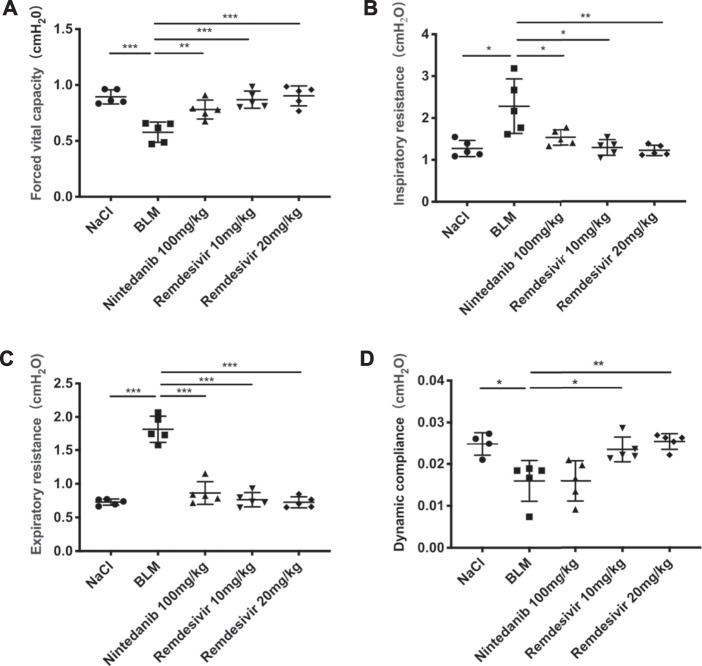
Remdesivir improves pulmonary function in BLM-treated mice. **(A)** Forced vital capacity of mice. **(B)** Inspiratory resistance of mice. **(C)** Expiratory resistance of mice. **(D)** Dynamic compliance of mice. Data was noted as the means ± SD, *n* = 5. * *p* < 0.05, ** *p* < 0.01, *** *p* < 0.001, **** *p* < 0.0001.

To determine the role of remdesivir in pulmonary fibrosis *in vivo*, C57BL/6 J mice were administered BLM (2 U), and then different doses of remdesivir (10 and 20 mg kg^−1^) were administered for 14 days (day 1-day 14) (Figure 1A). Nintedanib (100 mg kg^−1^) was used as a positive control. We found that the weight ratio, hydroxyproline level and percentage of fibrotic areas were significantly decreased in the remdesivir-treated group and that the inhibitory effect of remdesivir was better than that of nintedanib, while the survival rate of remdesivir was comparable to nintedanib (Figures 1B–E). Moreover, H and E and Masson’s trichrome staining were performed to assess the degree of pulmonary fibrosis. As shown in Figure 1F, BLM-induced collagen deposition was markedly reduced by remdesivir treatment. Lung function is a decisive factor in the diagnosis and treatment of pulmonary fibrosis in clinical trials. As shown in Figure 2, remdesivir significantly improved lung function in BLM-induced fibrotic mice, as seen by the increases in forced vital capacity (FVC) and dynamic compliance (Cdyn) and decreases in inspiratory resistance (Ri) and expiratory resistance (Re), and the effects were superior to those of the positive control drug nintedanib. These data revealed that remdesivir could ameliorate BLM-induced pulmonary fibrosis in mice.

### Remdesivir Suppresses TGF-β1-Induced Proliferation and Migration of Lung Fibroblasts

**FIGURE 3:**
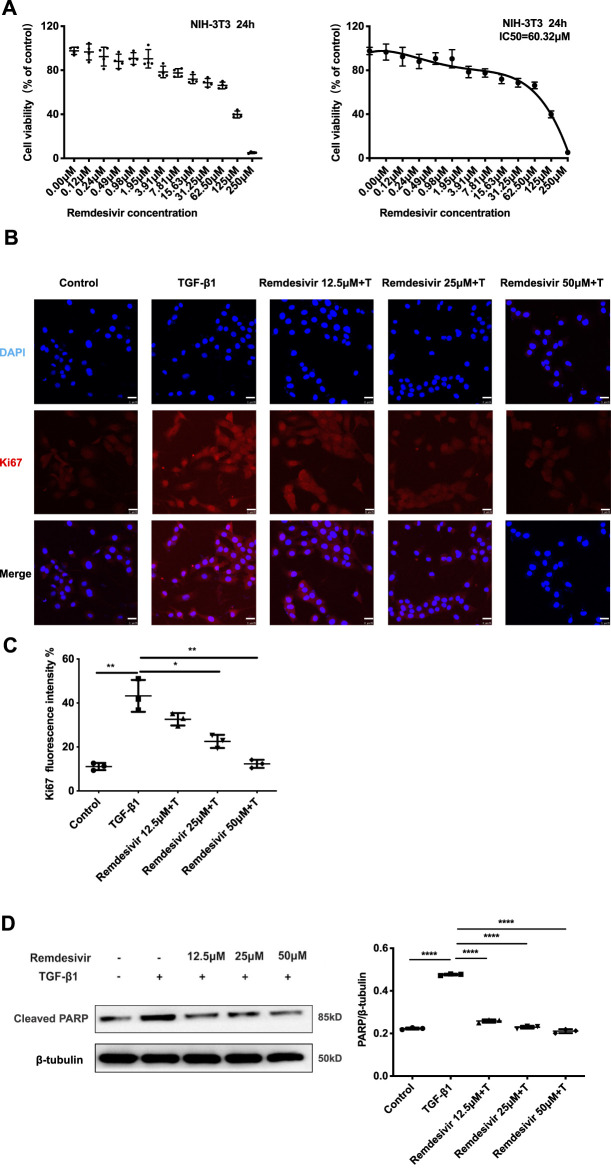
Remdesivir suppresses TGF-β1-induced proliferation of lung fibroblasts. **(A)** MTT assays of NIH-3T3 cells. Cells were exposed to the indicated doses of Remdesivir (0–250 μM) for 24 h, IC_50_ = 60.32 μM (n = 4 per group). **(B–C)** NIH-3T3 cells were co-treated with TGF-β1 (5 ng ml^−1^) and Remdesivir (12.5, 25, 50 μM) for 24 h to detect the Ki67 expression levels by immunofluorescence. The analyses of mean gray value were shown below. Scale bar = 60 μm (n = 3 per group). **(D)** NIH-3T3 cells were co-treated with TGF-β1 (5 ng ml^−1^) and Remdesivir (12.5, 25, 50 μM) for 24 h. Cleaved PARP were assessed using western blot. GAPDH was used as the internal control, *n* = 3. Data was presented as the means ± SD. *p < 0.05, **p < 0.01, ***p < 0.001, **** *p* < 0.0001.

The proliferation and migration of activated fibroblasts contribute to the development of lung fibrosis (Coward et al., 2010). First, we examined whether remdesivir affected the proliferation of normal fibroblasts using the MTT assay. We found that remdesivir had no obvious toxicity on normal fibroblasts, and the IC_50_ value of remdesivir in NIH-3T3 cells was 60.32 μM (Figure 3A). We also examined the effect of remdesivir on the cell cycle and senescence of normal fibroblasts in absence of TGF-β1 stimulation and the results showed that after treated with different doses of remdesevir, the fibroblasts didn’t arrest and becoming senescent (Supplementary Figure S1). To further investigate the effect of remdesivir on cell proliferation of active fibroblasts, we co-treated NIH3T3 cells with TGF-β1 (5 ng ml^−1^) and remdesivir (12.5, 25, and 50 μM), then used immunofluorescence and western blot to detect the expression of cell proliferation marker Ki67 and cell apoptosis maker cleaved PARP. As shown in Figures 3B–D, remdesivir could dose-dependently inhibit the proliferation and apoptosis of TGF-β1-activated fibroblasts.

**FIGURE 4:**
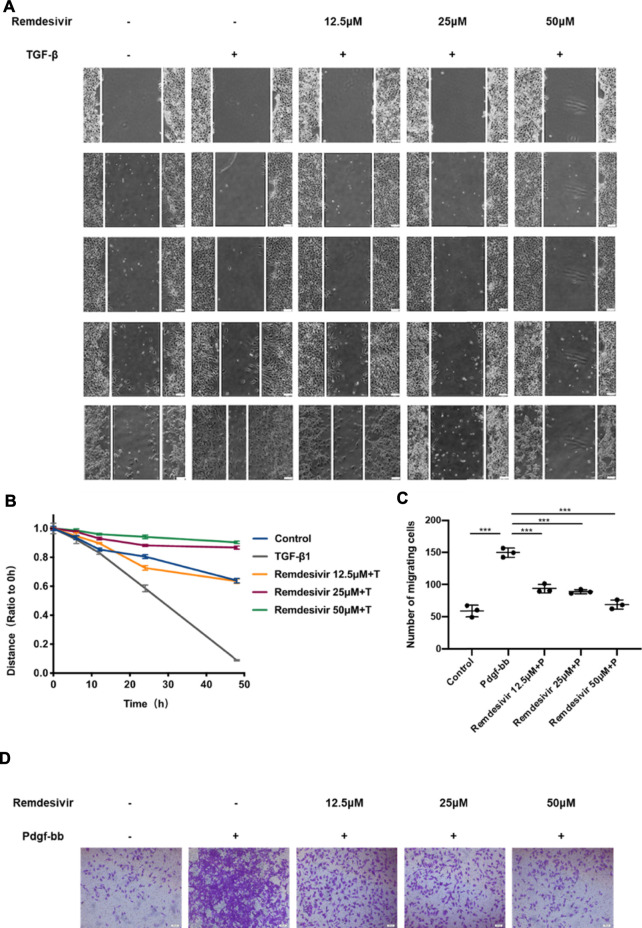
Remdesivir suppresses TGF-β1-induced migration of lung fibroblasts. **(A–B)** Wound healing assays of NIH3T3 co-cultured with TGF-β1 (5 ng ml^−1^) and Remdesivir (12.5, 25, 50 μM). The wound closure was photographed at 0, 6, 12, 24, and 48 h post-scratching **(C–D)** NIH-3T3 cells treated with/without Remdesivir (12.5, 25, 50 μM) and/or Pdgf-bb (5 ng ml^−1^). Scale bar = 60 μm. Data was presented as the means ± SD, *n* = 3. * *p* < 0.05, ** *p* < 0.01, *** *p* < 0.001, **** *p* < 0.0001.

Then, we used wound healing assay and transwell assay to test the effects of remdesivir on the migration of activated fibroblasts. NIH-3T3 cells were co-treated with remdesivir (12.5, 25, and 50 μM) and TGF-β1 (5 ng ml^−1^) for 6, 12, 24, and 48 h. As shown in Figures 4A–C, remdesivir dose-dependently inhibited the migration of TGF-β1-activated fibroblasts. The transwell assay also indicated that remdesivir could dose-dependently inhibit PDGF-induced migration of fibroblasts (Figure 4D).

### Remdesivir Attenuates TGF-β1-Induced Activation of Lung Fibroblasts

**FIGURE 5:**
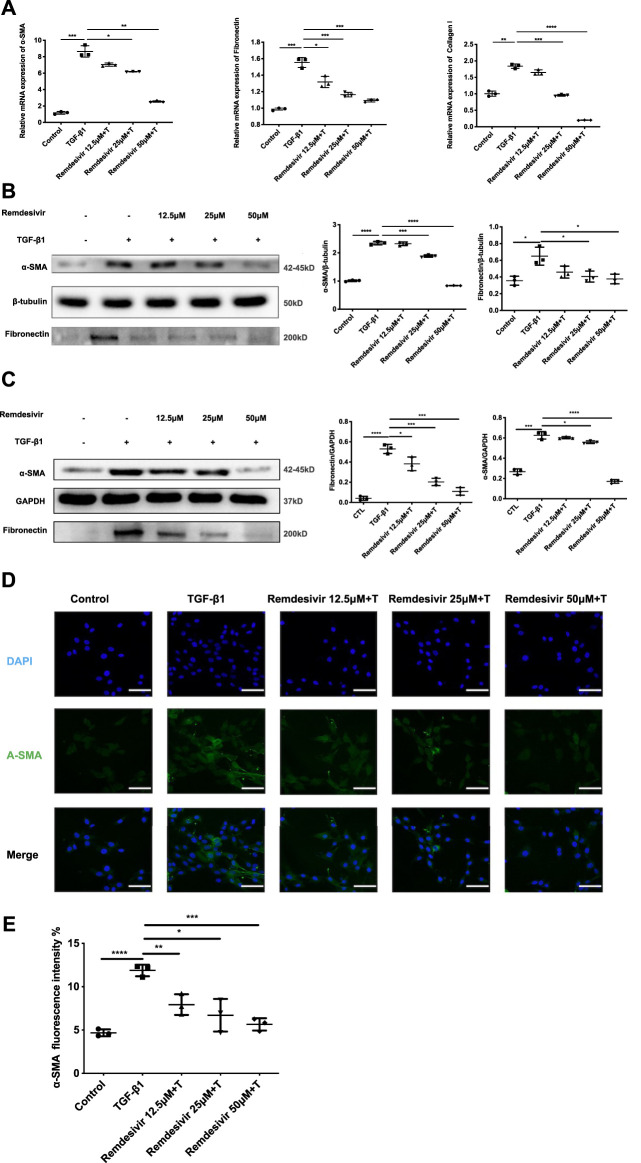
Remdesivir attenuates TGF-β1-induced activation of lung fibroblasts. **(A)** NIH-3T3 cells were co-treated with TGF-β1 (5 ng ml^−1^) and Remdesivir (12.5, 25, 50 μM) for 24 h. mRNA levels of α-SMA, Fibronectin and Collagen I were tested by RT-PCR in NIH-3T3 cells **(B)** NIH-3T3 cells were co-treated with TGF-β1 (5 ng ml^−1^) and Remdesivir (12.5, 25, 50 μM) for 24 h. α-SMA and Fibronectin were assessed using western blot, GAPDH was used as the internal control **(C)** PPF cells were co-treated with TGF-β1 (5 ng ml^−1^) and Remdesivir (12.5, 25, 50 μM) for 24 h. α-SMA and Fibronectin were assessed using western blot, β-tubulin was used as the internal control **(D)** Immunofluorescence staining of α-SMA were performed on NIH-3T3 cells treated with/without TGF-β1 (5 ng ml^−1^) and/or Remdesivir (12.5, 25, 50 μM) for 24 h. Data was noted as the means ± SD, *n* = 3. *p < 0.05, **p < 0.01, ***p < 0.001, **** *p* < 0.0001.

To further examine whether remdesivir could inhibit the activation of TGF-β1-induced fibroblasts, we treated NIH-3T3 cells and PPF cell with TGF-β1 (5 ng ml^−1^) and remdesivir (12.5, 25, and 50 μM) to assess the gene expression levels of the typical fibroblast activation marker α-SMA and the extracellular matrix (ECM) proteins collagen I and fibronectin. As shown in Figure 5A, remdesivir dose-dependently decreased TGF-β1-induced expression of the α-SMA, collagen I and fibronectin genes. Similarly, remdesivir treatment reduced the protein expression levels of α-SMA, and fibronectin in TGF-β1-stimulated fibroblasts, including NIH3T3 cell and PPF cell (Figures 5B,C). Immunofluorescence assays were used to evaluate α-SMA expression in NIH-3T3 cells, and the results were consistent (Figures 5D,E). Accordingly, these results indicated that remdesivir suppressed TGF-β1-induced myofibroblast differentiation and reduced ECM production in myofibroblasts.

### Remdesivir Inhibits TGF-β1-Induced Activation of Smad and Non-smad Signaling Pathways in Lung Fibroblasts

**FIGURE 6:**
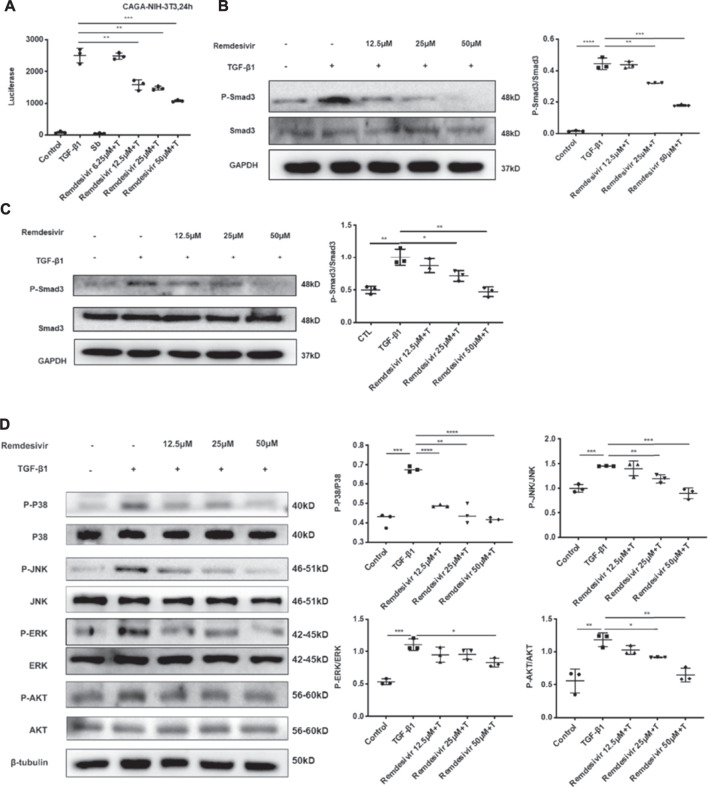
Remdesivir inhibits TGF-β1-induced activation of Smad and non-Smad signaling pathway in lung fibroblasts **(A)** Luciferase assays of CAGA-NIH3T3 cells. Cells were pretreated with Remdesivir (0–50 μM) for 30 min and then incubated with TGF-β1 (5 ng ml^−1^) for 24 h, then analyzed with luciferase assay. SB431542 is a TGF-β1/Smad pathway inhibitor and serves as a positive control **(B)** NIH-3T3 cells were co-treated with TGF-β1 (5 ng ml^−1^) and Remdesivir (12.5, 25, 50 μM) for 1 h. P-Smad3 and Smad3 were assessed using western blot. GAPDH was used as the internal control **(C)** PPF cells were co-treated with TGF-β1 (5 ng ml^−1^) and Remdesivir (12.5, 25, 50 μM) for 1 h. P-Smad3 and Smad3 were assessed using western blot. GAPDH was used as the internal control (D) NIH-3T3 cells were co-treated with TGF-β1 (5 ng ml^−1^) and Remdesivir (12.5, 25, 50 μM) for 1 h and the phosphorylation levels of P-38, JNK, ERK and Akt were analyzed by Western blot. β-tubulin was used as a loading control in grayscale analysis. Scale bar = 60 μm. Data was presented as the means ± SD, *n* = 3. * *p* < 0.05, ** *p* < 0.01, *** *p* < 0.001, **** *p* < 0.0001.

The TGF-β1 signaling pathway is critical in regulating the proliferation and activation of fibroblasts in pulmonary fibrosis (Aschner and Downey, 2016). Therefore, we examined whether remdesivir could regulate TGF-β1/Smad and non-Smad signaling in active pulmonary fibroblasts. We stably transfected the (CAGA)_12_-luciferase reporter, which contain repeated canonical Smad3-binding element (SBE) 5′-CAGA-3′ in NIH-3T3 fibroblasts. CAGA-NIH-3T3 cells were used to examine the inhibitory effects of compounds on the TGF-β/Smad3 signaling pathway. After the administration of 5 ng ml^−1^ TGF-β1, the fluorescence value was obviously increased in CAGA-NIH-3T3 cells, while remdesivir inhibited fluorescence in a dose-dependent manner (Figure 6A). Next we used the western blot assay to detect the effect of remdesivir on the activation of TGF-β1/Smad and non-Smad signaling in fibroblasts. We first examined the effect of TGF-β1 on the protein phosphorylation level of Smad and non-Smad under the time gradient, and the results revealed that the phosphorylation level of the protein was positively correlated with the time of adding TGF-β1 and the phosphorylation levels were more significant after 1 h of TGF-β stimulation (Supplementary Figure S2). Then we assessed the effect of remdesivir on the activation of Smad3 signaling in NIH-3T3 cells and PPF cells after 1 h treatment of TGF-β1. As shown in Figures 6B,C, remdesivir significantly reduced the phosphorylation level of Smad3 and had no significant effect on the protein levels of total Smad3. Moreover, the levels of phosphorylated P38, JNK, ERK and Akt were examined in NIH-3T3 cells by western blotting. The results revealed that remdesivir restrained the activation of TGF-β1/Smad and non-Smad signaling (Figure 6D).

### Remdesivir Inhibits TGF-β1-Induced Phenotypic Transition in Alveolar Epithelial Cells

**FIGURE 7:**
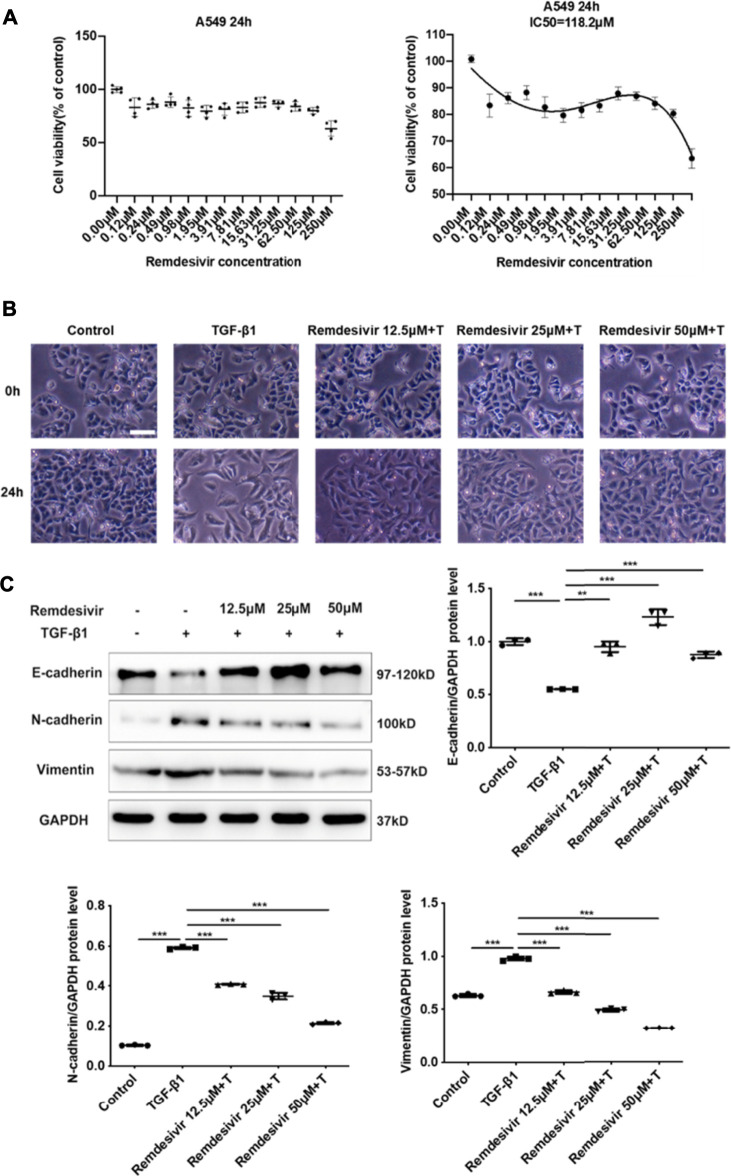
Remdesivir inhibits TGF-β1-induced epithelial to mesenchymal transition in alveolar epithelial cells **(A)** MTT assays of A549 cells. Cells were exposed to the indicated doses of Remdesivir (0–250 μM) for 24 h, IC_50_ = 118.2 μM (*n* = 4 per group) **(B–C)** A549 cells were co-treated with Remdesivir (12.5, 25, 50 μM) and TGF-β1 (5 ng ml^−1^) for 24 h **(B)** Morphological changes of A549 cells. **(C)** Protein expression levels of E-cadherin, Vimentin and N-cadherin were assessed by Western blot in A549 cells. GAPDH was used as a loading control. Data was presented as the means ± SD, *n* = 3. * *p* < 0.05, ** *p* < 0.01, *** *p* < 0.001, **** *p* < 0.0001.

**FIGURE 8:**
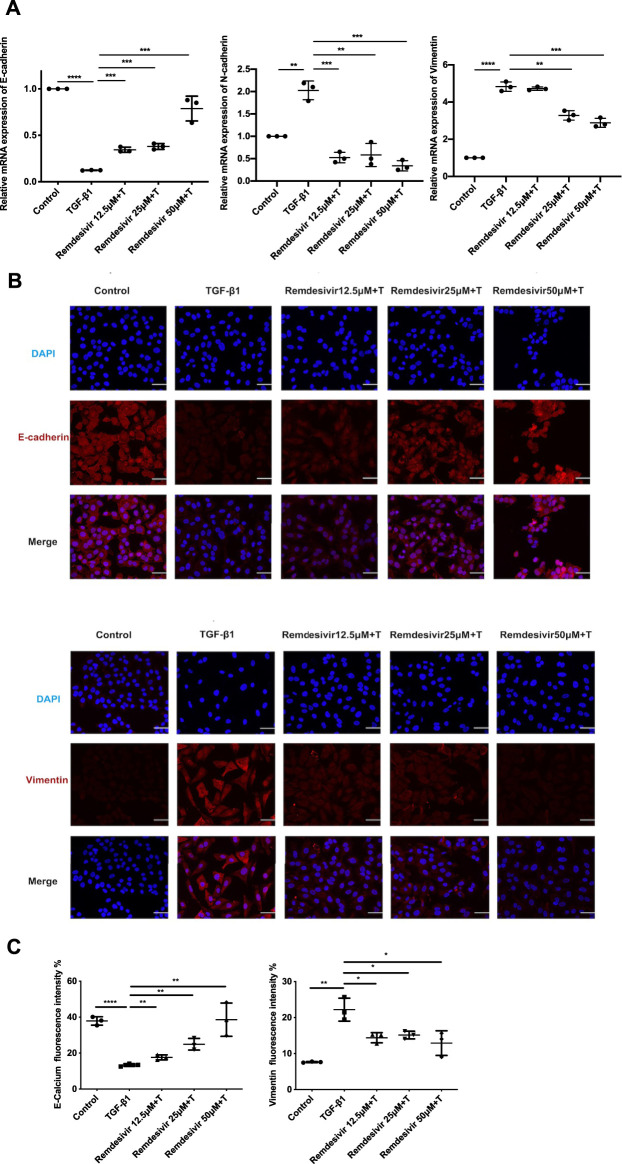
Remdesivir inhibits TGF-β1-induced epithelial to mesenchymal transition in alveolar epithelial cells. A549 cells were co-treated with Remdesivir (12.5, 25, 50 μM) and TGF-β1 (5 ng ml^−1^) for 24 h **(A)** mRNA levels of E-cadherin, N-cadherin and Vimentin were tested by RT-PCR in A549 cells. **(B–C)** Immunofluorescence analysis of E-cadherin and Vimentin in A549 cells. Scale bar = 50 μm. Data was presented as the means ± SD, *n* = 3. * *p* < 0.05, ** *p* < 0.01, *** *p* < 0.001.

Evidence suggests that lung fibrosis is a dysregulated epithelial-mesenchymal disorder in which epithelial injury is an initiating event (Selman and Pardo, 2002). The hallmark features of injured epithelial cells are the decreased expression of epithelial markers, including E-cadherin, and the increased expression of mesenchymal markers, such as N-cadherin and vimentin (King et al., 2011; Kage and Borok, 2012). First, we examined whether remdesivir affected the proliferation of A549 cells using the MTT assay. We found that remdesivir had no obvious toxicity on A549 cell, and the IC_50_ value of remdesivir in A549 cells was 118.2 μM (Figure 7A). To explore whether remdesivir exerted antifibrotic effects by protecting against epithelial to mesenchymal transition, we used stimulated human pulmonary epithelial cells (A549 cells) with TGF-β1 and then treated the cells with remdesivir (12.5, 25, and 500 μM). After 24 h, the morphological changes and expression of epithelial/mesenchymal phenotypic markers in A549 cells were examined. The results indicated that remdesivir could effectively reverse the morphological changes in TGF-β1-induced A549 cells (Figure 7B). The qRT-PCR and western blot results indicated that remdesivir could significantly increase the expression of the epithelial marker E-cadherin and decrease the expression of the mesenchymal markers N-cadherin and vimentin in A549 cells (Figure 7C; Figure 8A). Moreover, the immunofluorescence staining results were consistent with the above results (Figures 8B,C). Collectively, these data indicate that remdesivir inhibits TGF-β1-induced phenotypic transition in alveolar epithelial cells.

### Remdesivir Attenuates BLM-Induced Fibroblast Activation and Epithelial to Mesenchymal Transition *in vivo*

**FIGURE 9:**
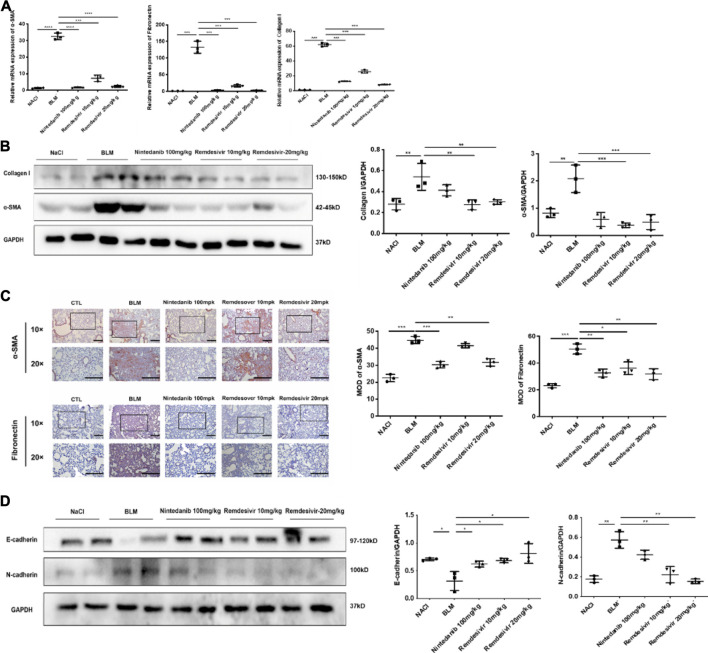
Remdesivir attenuates BLM-induced fibroblast activation and epithelial injury *in vivo* **(A)** RT-PCR was performed to detect mRNA levels of α-SMA, Fibronectin and Collagen I in lung tissues. **(B)** Protein levels of α-SMA and Collagen I were verified by Western blot in lung tissues. GAPDH was used as an internal reference in densitometric analysis **(C)** Immunohistochemical staining analysis of α-SMA and Fibronectin in lung tissues. **(D)** Protein expression levels of E-cadherin and N-cadherin were assessed by Western blot in lung tissues. GAPDH was used as a loading control. Data was presented as the means ± SD, *n* = 3. * *p* < 0.05, ** *p* < 0.01, *** *p* < 0.001.

**FIGURE 10:**
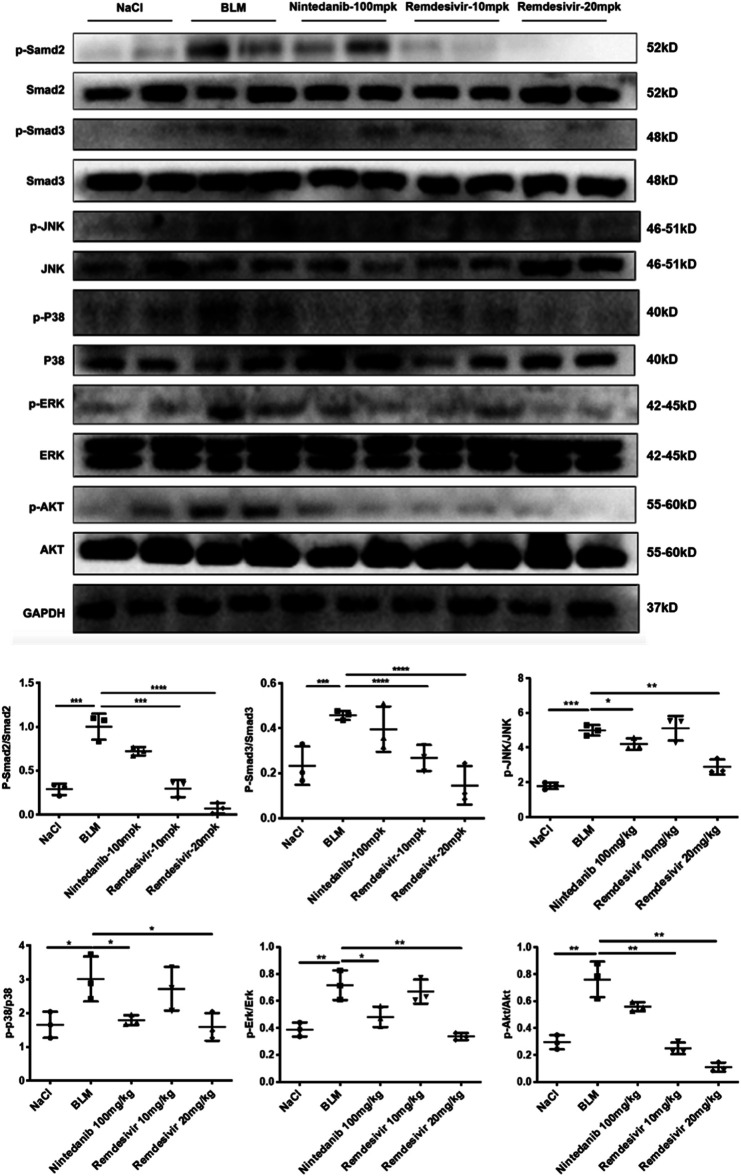
Remdesivir suppress BLM-induced pulmonary fibrosis in mice via inhibiting TGF-β1-Smad and non-Smad signaling pathway *in vivo*. Protein levels of p-Smad2, Smad2, p-Smad3, Smad3, p-P38, P38, p-JNK, JNK, p-ERK, ERK, p-AKT, and AKT were verified by Western blot in lung tissues. GAPDH was used as an internal reference in densitometric analysis. Data was presented as the means ± SD, *n* = 3. ** *p* < 0.01, *** *p* < 0.001.

To further verify whether remdesivir could reduce the activation of lung fibroblasts *in vivo*, we extracted protein and RNA from lung tissues and analyzed them by western blotting and qRT-PCR. The results confirmed that the protein and RNA expression levels of α-SMA and collagen I in the lung tissues of the remdesivir group were significantly lower than those in the BLM model group and better than those of the positive control group (Figures 9A,B). We also performed immunohistochemical analysis of lung tissue sections, and the expression levels of α-SMA and fibronectin in the lung tissues of remdesivir-treated mice were lower than those in the BLM model group (Figure 9C). Epithelial-mesenchymal transition markers were evaluated, and similar to our *in vitro* results, western blotting showed that E-cadherin expression was increased and N-cadherin expression was decreased by remdesivir treatment (Figure 9D). Then we further verified whether remdesivir could influence TGF-β1 signaling pathway *in vivo*, the western blotting results indicated that remdesivir significantly reduced the activation of Smad2/3 and MAPK signaling in the lung tissues of bleomycin-injured mice (Figure 10). In conclusion, remdesivir attenuated BLM-induced fibroblast activation and epithelial to mesenchymal transition *in vivo*.

## Discussion

In this study, we first reported that remdesivir could attenuate BLM-induced pulmonary fibrosis by suppressing the proliferation, migration and activation of lung fibroblasts and improving alveolar epithelial to mesenchymal transition. We confirmed that TGF-β1 signaling is the key pathway in the antifibrotic pharmacological mechanism of remdesivir. Thus, we propose that remdesivir may be a potential drug candidate for preventing pulmonary fibrosis.

In the past year, SARS-CoV-2 has spread around the globe, infecting millions and claiming far too many lives. According to a case report, 25.9% of COVID-19 patients were seriously ill, and 17.2–31% of COVID-19 patients suffered from viral-induced ARDS (Lai et al., 2020). A considerable proportion of ARDS patients will suffer from long-term damage to lung function, and CT follow-up of these patients also showed evidence of pulmonary fibrosis (Herridge et al., 2011; Masclans et al., 2011). The pathology of severe COVID-19-related lung damage also indicated the existence of pulmonary fibrosis, which was shown as hyperplastic and metaplastic changes in pneumocytes and interstitial collagen fiber deposits (Kommoss et al., 2020). In recent decades, a single tracheal instillation of BLM in rodents has been a widely used model for studying the occurrence of pulmonary fibrosis and evaluating the effects of antifibrotic treatments (Jenkins et al., 2017). BLM induces a significant fibrotic reaction in the lungs, and the pathological manifestations are similar to clinical manifestations, including destruction of alveolar structure, abnormal fibroblast proliferation and differentiation, and excessive deposition of ECM proteins (Kolb et al., 2020). The initiation of BLM-induced injury is also an acute inflammatory response, including the infiltration of inflammatory cells and the upregulation of acute inflammatory factors such as interleukins, and the disease will then progress to pulmonary fibrosis (Chaudhary et al., 2006). In our study, we used a BLM-induced model to mimic post-COVID-19 fibrosis and evaluated the effect of remdesivir on pulmonary fibrosis. The results indicated that remdesivir could attenuate BLM-induced collagen deposition, improve lung function *in vivo*, and inhibit myofibroblast activation and epithelial injury both *in vivo* and *in vitro*.

**FIGURE 11:**
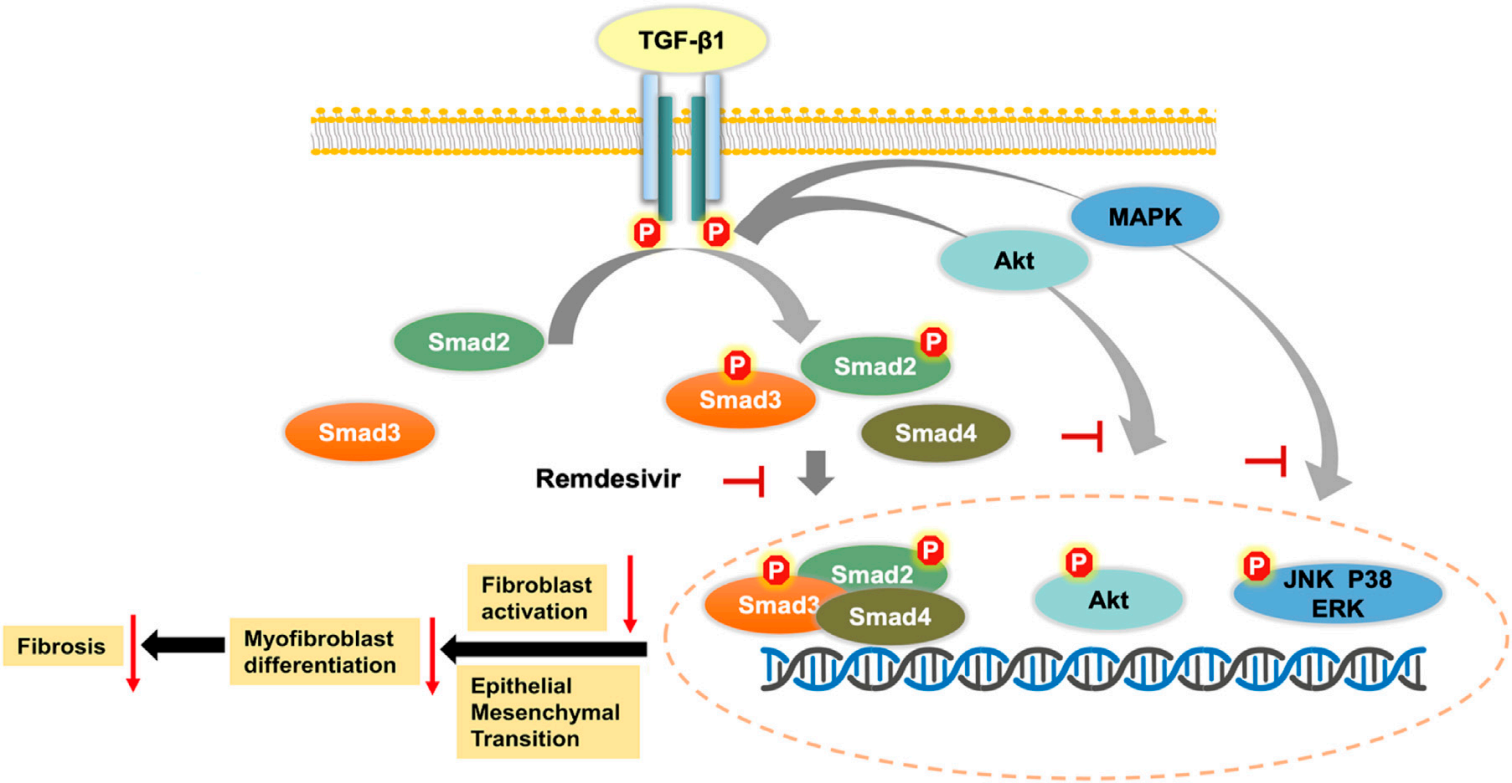
Mechanism for the anti-pulmonary fibrosis effect of remdesivir.

TGF-β is a widely expressed multifunctional cytokine that plays a key role in the tissue repair process after injury. TGF-β1 is the core regulatory factor in the pathological repair process associated with pulmonary fibrosis (Aschner and Downey, 2016). In the pathological process of pulmonary fibrosis, TGF-β1 activates Smad and non-Smad transcription factors via the serine-threonine kinase transduction pathway and then promotes the transcription of genes involved in ECM formation, stimulates fibroblast migration and proliferation, induces the deposition of collagen and fibronectin, and promotes alveolar epithelial cell injury (Fernandez and Eickelberg, 2012). Here, we first demonstrated that remdesivir attenuated pulmonary fibrosis by inhibiting the TGF-β signaling pathway. The *in vitro* data indicated that remdesivir suppressed TGF-β1-induced proliferation, migration, and activation of lung fibroblasts and improved TGF-β1-induced alveolar epithelial cell injury by inhibiting the TGF-β1/Smad and TGF-β1/non-Smad signaling pathways (Figure 11). These data indicate that in addition to antiviral targets, remdesivir may interfere with the TGF-β1 pathway through other targets, and these specific targets will be further studied.

## Data Availability

The original contributions presented in the study are included in the article/Supplementary Material, further inquiries can be directed to the corresponding author.
