# Supplementary material for: Protective Effect of Remdesivir Against Pulmonary Fibrosis in Mice
Source: Front Pharmacol. 2021 Aug 26;12:692346. doi: 10.3389/fphar.2021.692346 (PMC8427522; doi:10.3389/fphar.2021.692346)
Supplement: Supplementary file 1 [file DataSheet3.docx]

**
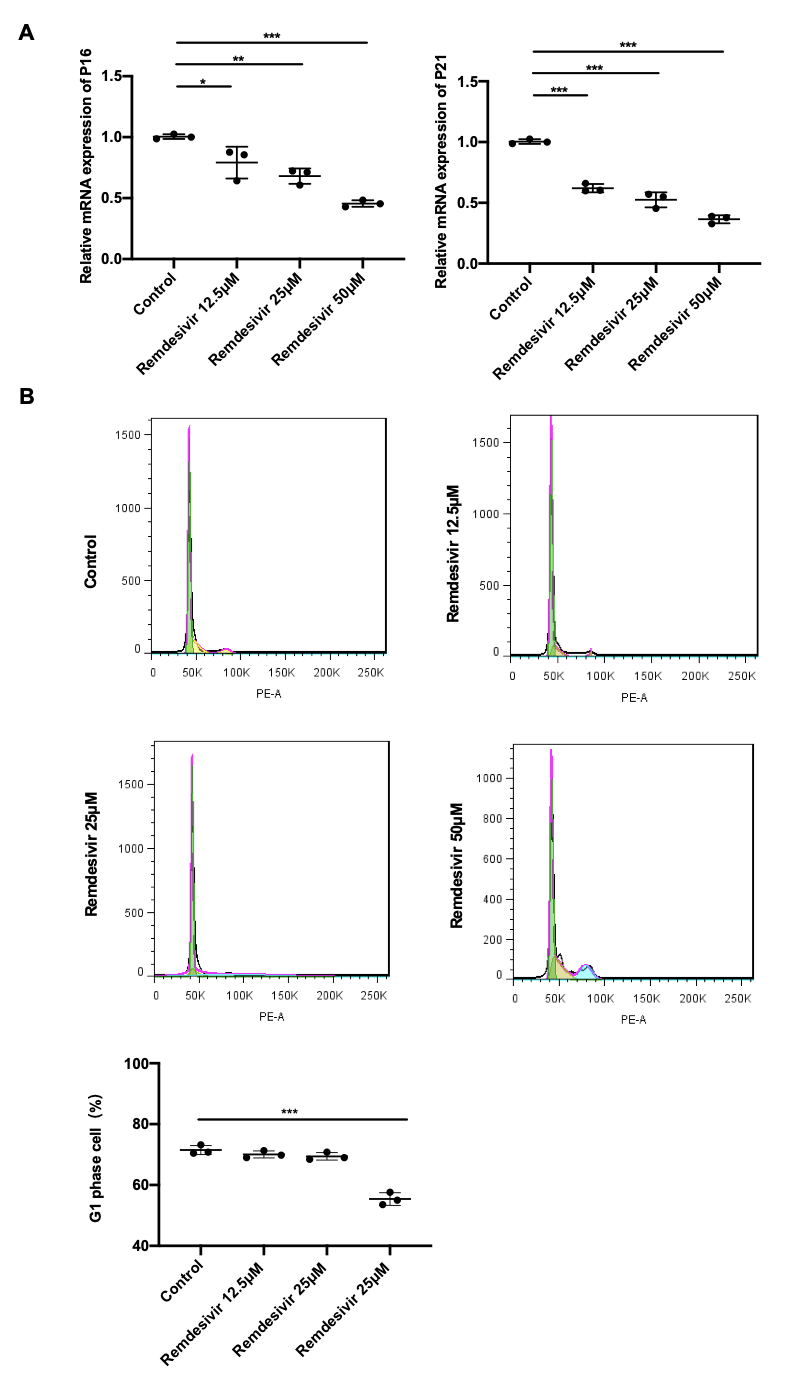
**

**Fig.S1. The effect of Remdesivir on cell cycle and aging of normal fibroblasts.** (A) NIH-3T3 cells were treated with Remdesivir (12.5 μM, 25 μM, 50 μM) for 24h. RT-PCR was performed to detect mRNA levels of P16 and P21. (B) NIH-3T3 cells were treated with/without Remdesivir (12.5 μM, 25 μM, 50 μM) for 24 h and then cell cycle was detected by flow cytometry. Data was presented as the means ± SD, n = 3. * P<0.05, ** P<0.01, *** P<0.001.


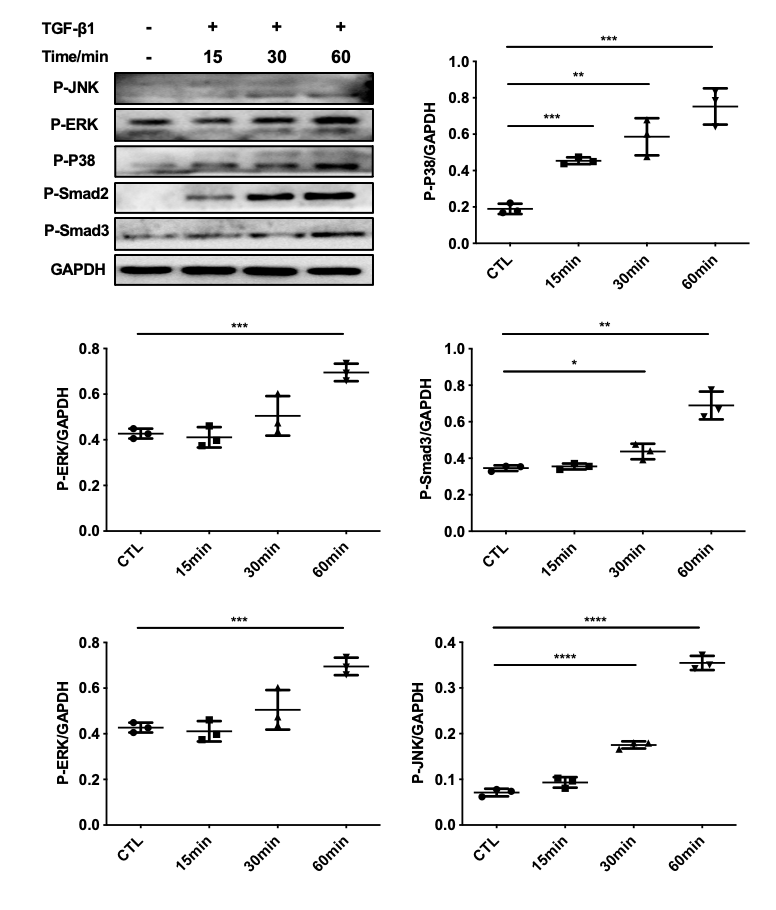


**Fig.S2. The phosphorylation level of Smad/non-Smad proteins after time gradient stimulation of TGF-β1 in lung fibroblasts.** NIH-3T3 cells were treated with TGF-β1 (5 ng·ml−1) for 0, 15, 30 and 60 min, then the phosphorylation levels of JNK, ERK, P38, Smad2 and Smad3 were analyzed by Western blot. GAPDH was used as a loading control in grayscale analysis. Data was noted as the means ± SD, n = 3. *P < 0.05, **P < 0.01, ***P < 0.001, **** P<0.0001.
