# Supplementary figures and images for: Protective Effect of Remdesivir Against Pulmonary Fibrosis in Mice
Source: Front Pharmacol. 2021 Aug 26;12:692346. doi: 10.3389/fphar.2021.692346 (PMC8427522; doi:10.3389/fphar.2021.692346)

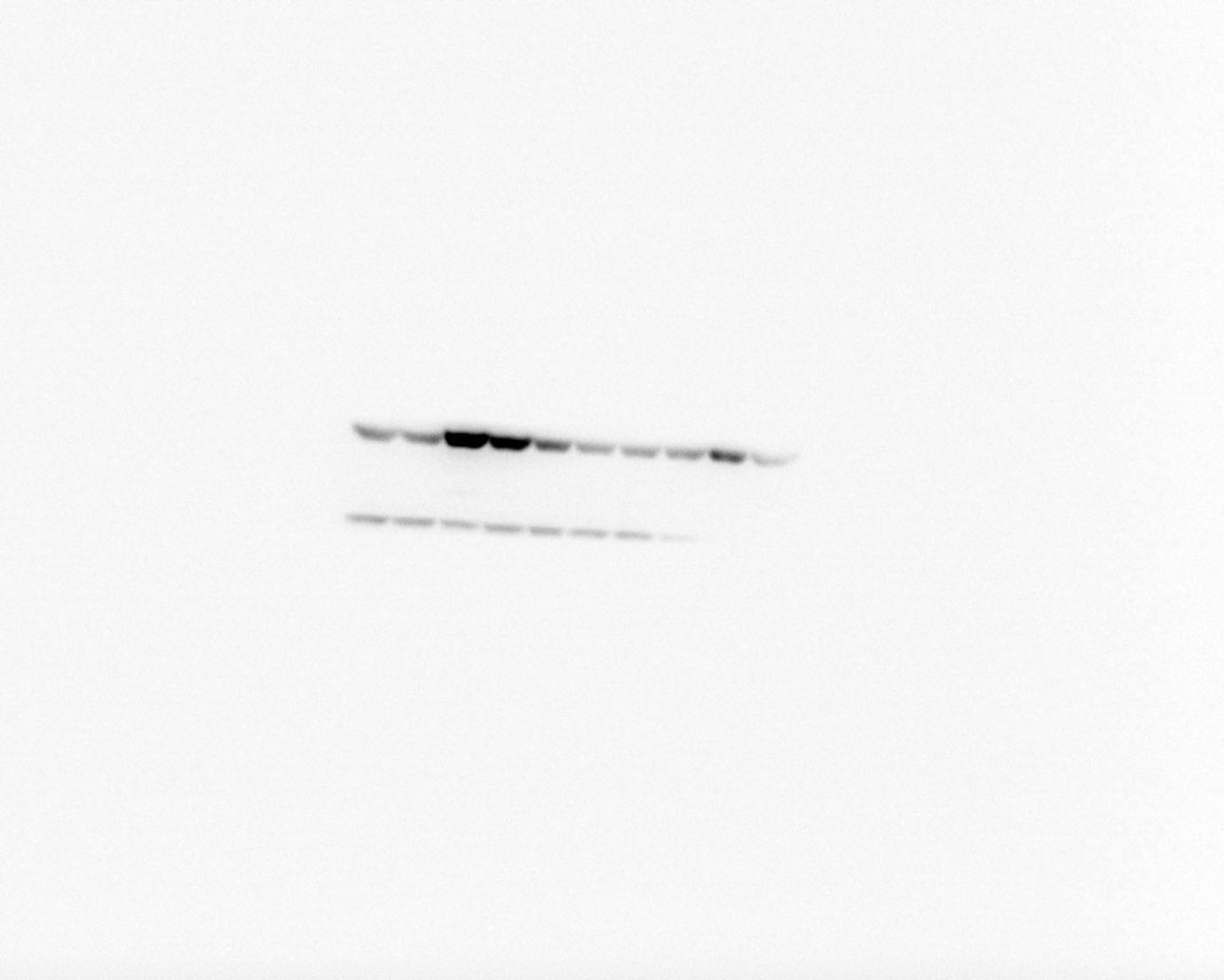

Supplement: Supplementary file 2 [file DataSheet1.ZIP › original blot images-Fig5-9/Fig9B-╬▒-sma.tif]

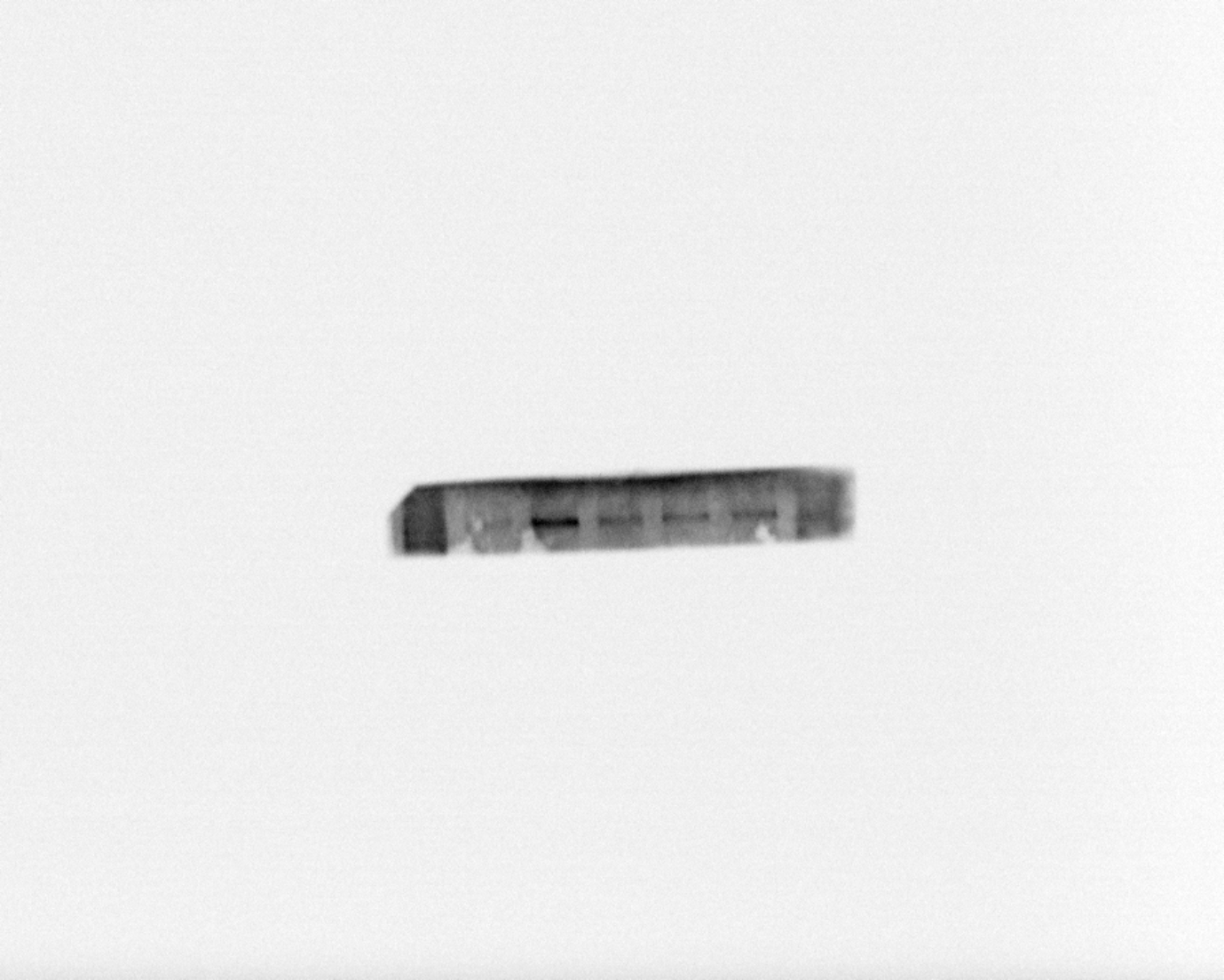

Supplement: Supplementary file 2 [file DataSheet1.ZIP › original blot images-Fig5-9/Fig5B-Fn.tif]

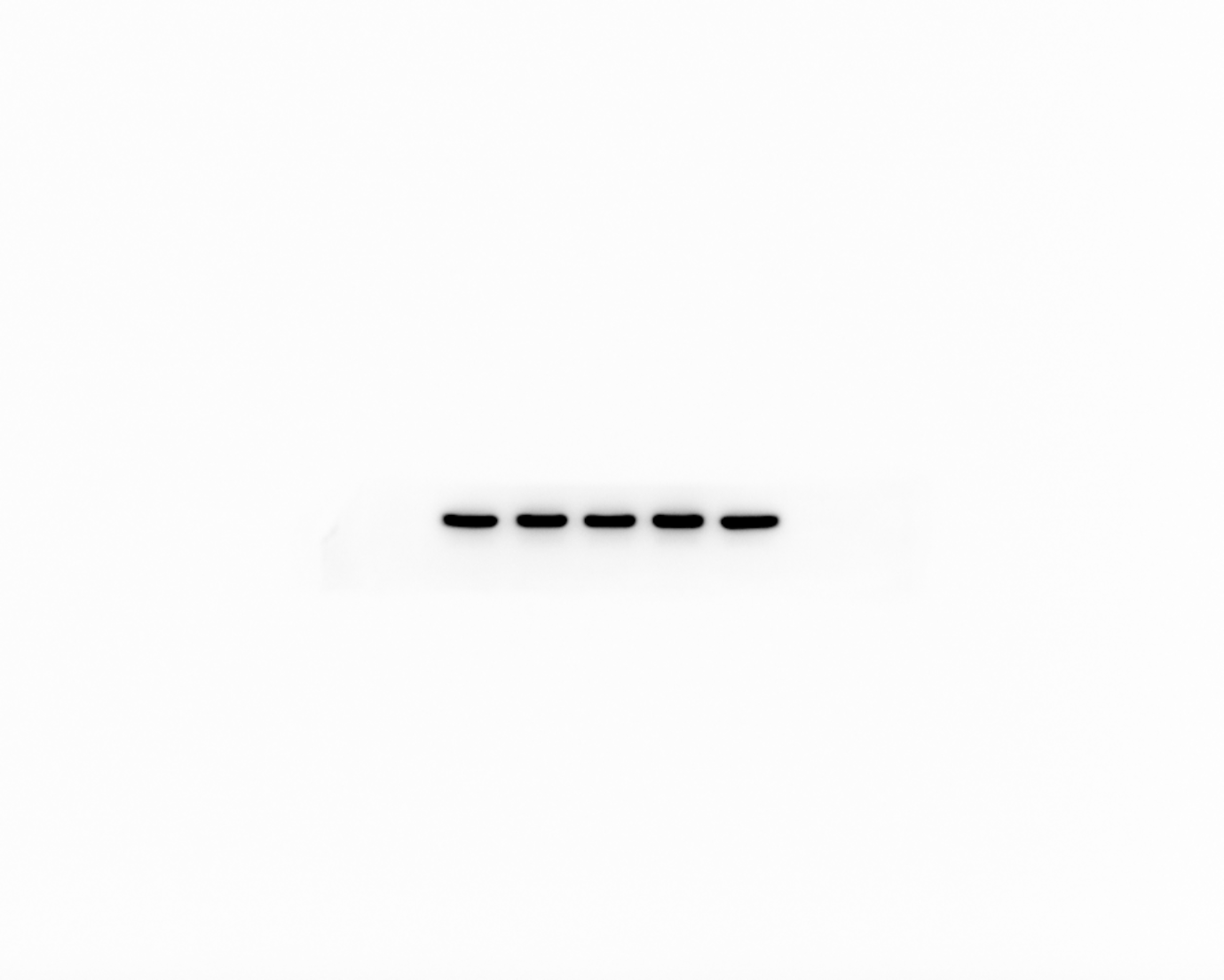

Supplement: Supplementary file 2 [file DataSheet1.ZIP › original blot images-Fig5-9/Fig7C-GAPDH.tif]

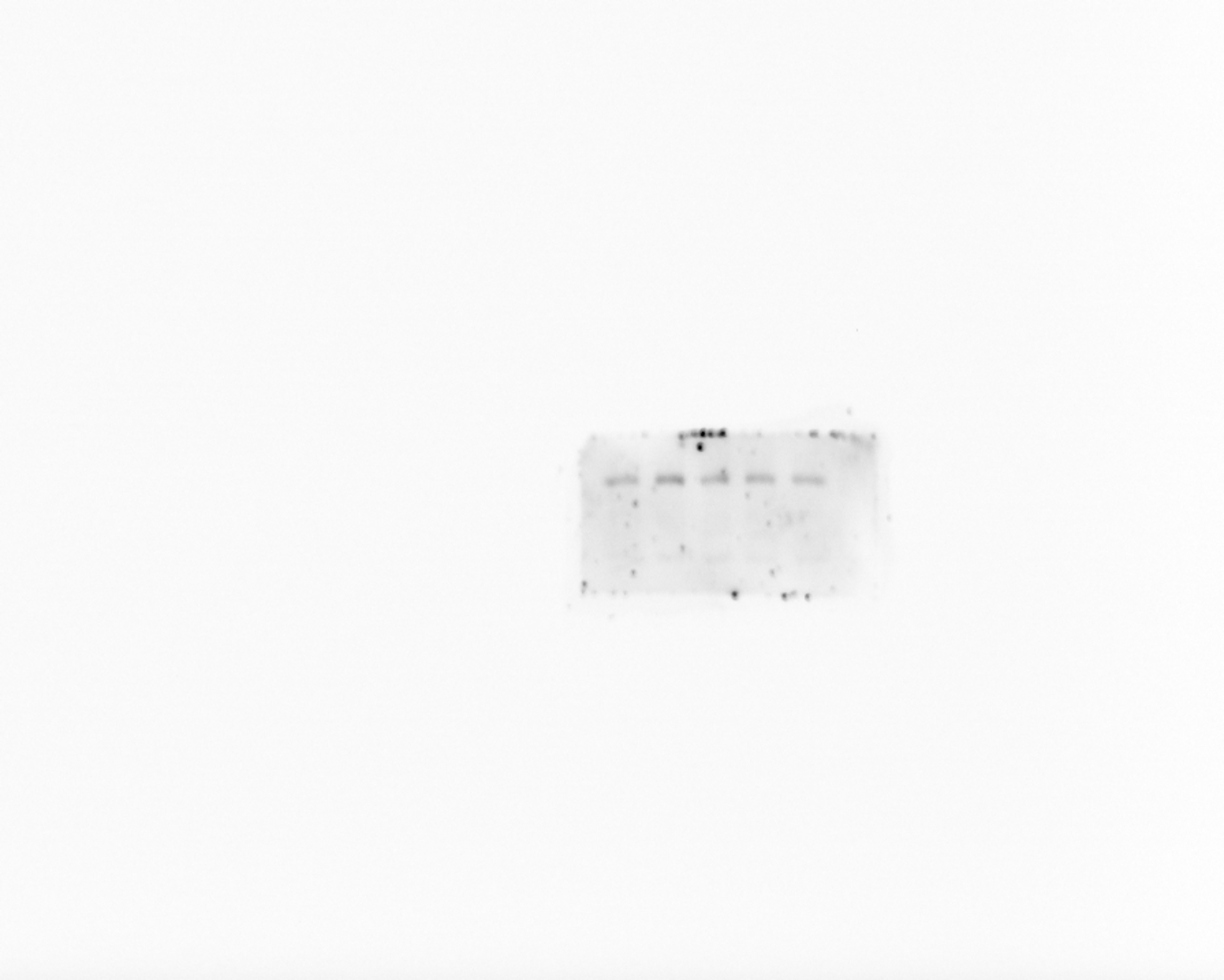

Supplement: Supplementary file 2 [file DataSheet1.ZIP › original blot images-Fig5-9/Fig6D-P-AKT.tif]

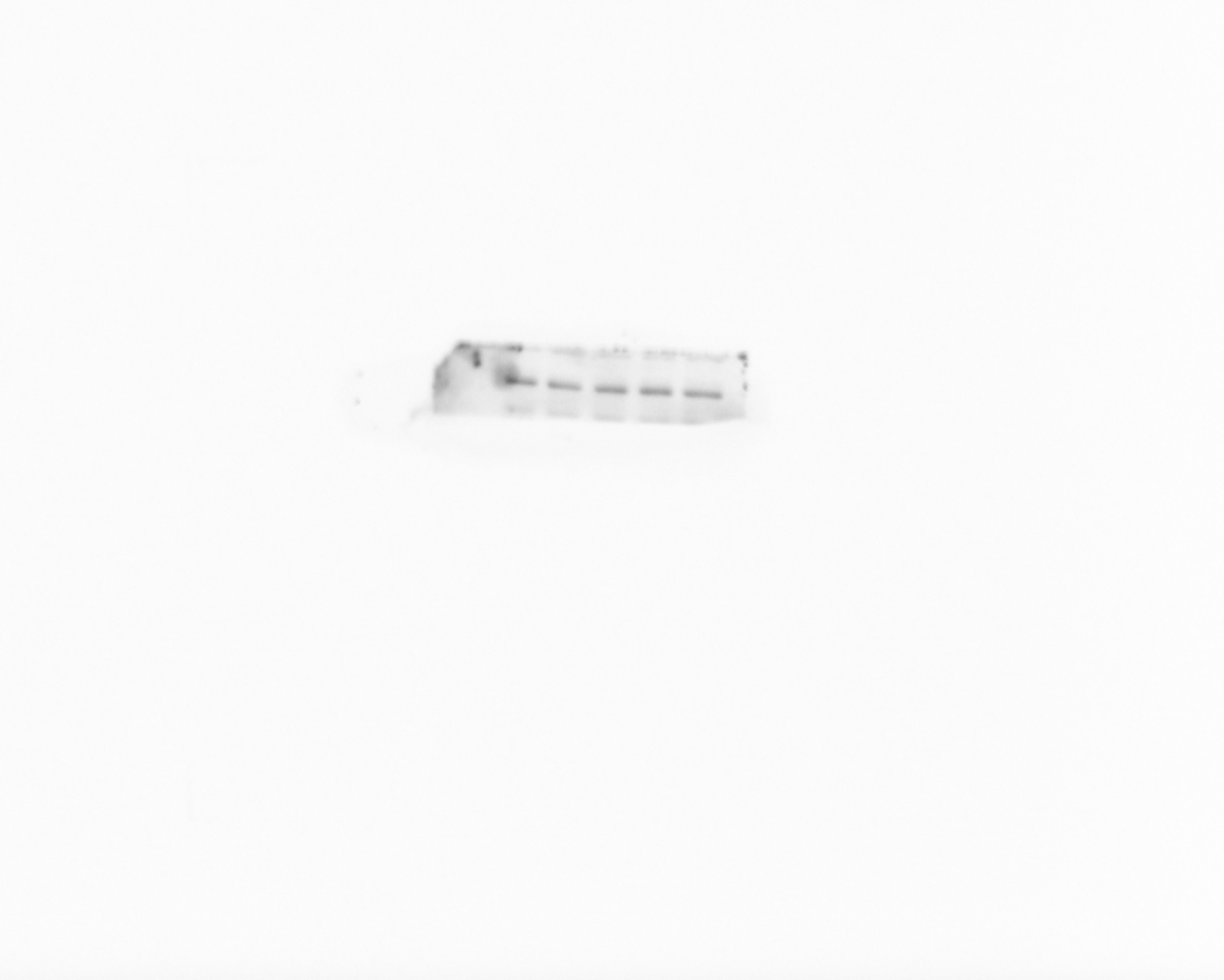

Supplement: Supplementary file 2 [file DataSheet1.ZIP › original blot images-Fig5-9/Fig6D-AKT.tif]

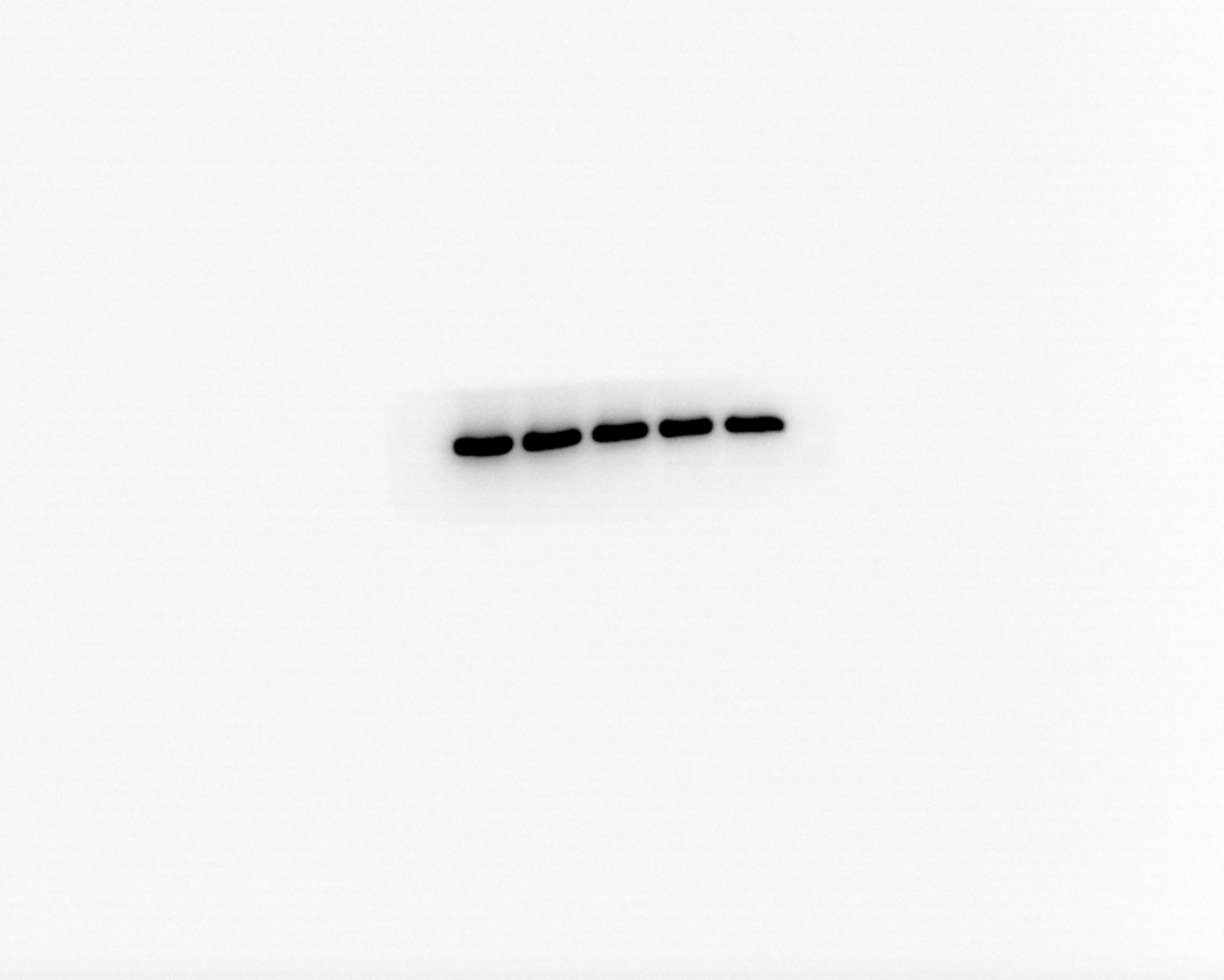

Supplement: Supplementary file 2 [file DataSheet1.ZIP › original blot images-Fig5-9/Fig5C-GAPDH.tif]

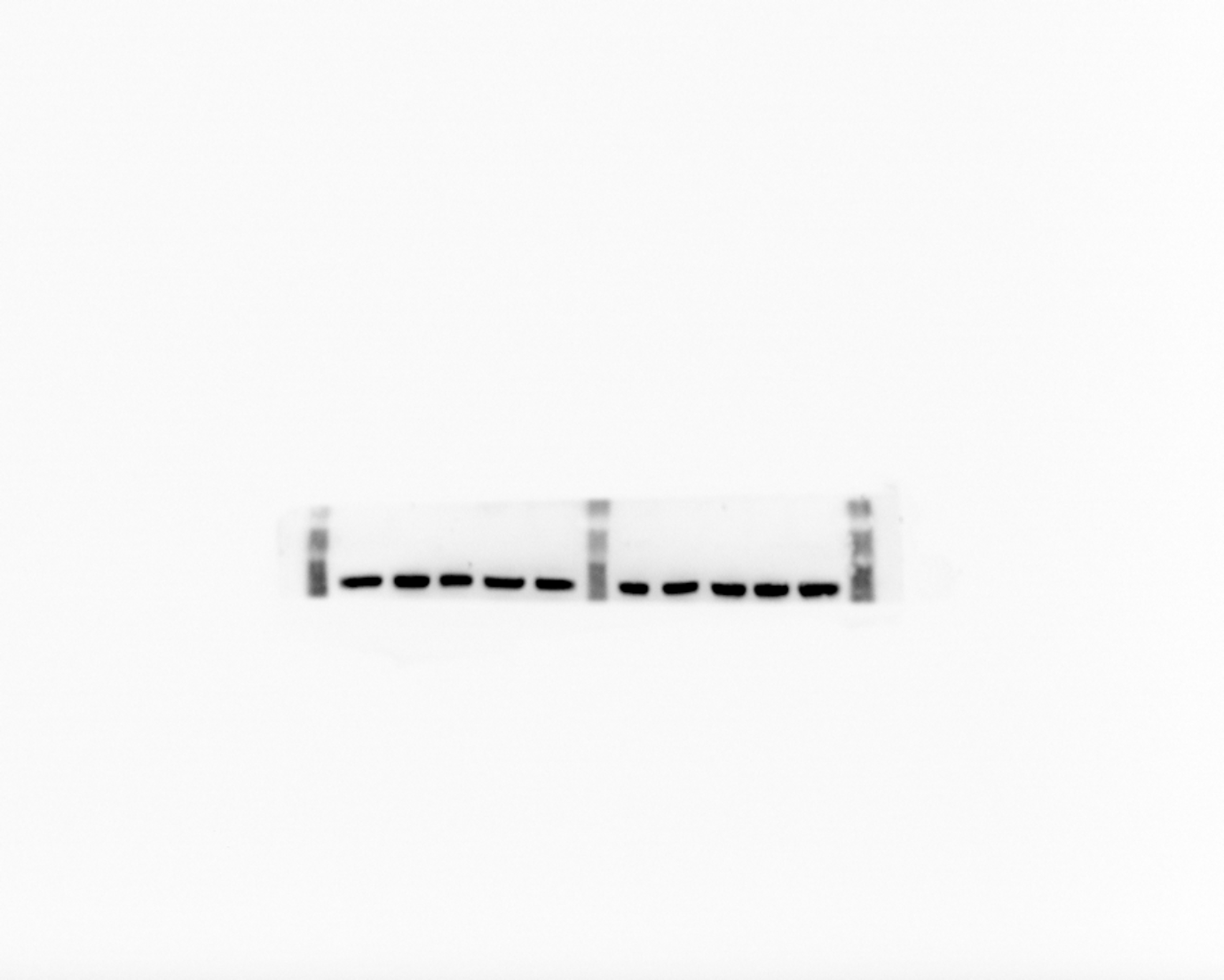

Supplement: Supplementary file 2 [file DataSheet1.ZIP › original blot images-Fig5-9/Fig6D-╬▓-tubulin.tif]

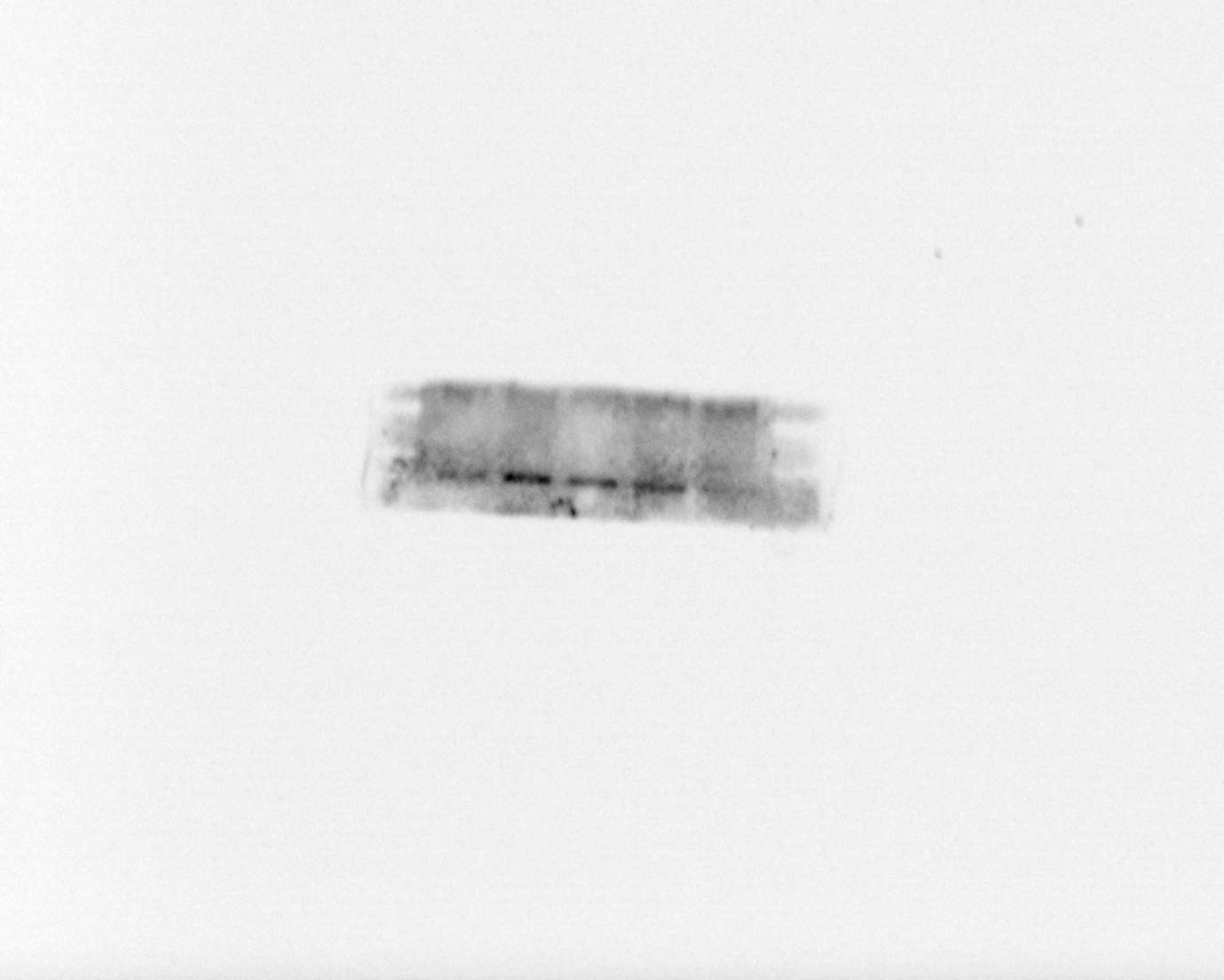

Supplement: Supplementary file 2 [file DataSheet1.ZIP › original blot images-Fig5-9/Fig6C-p-Smad3.tif]

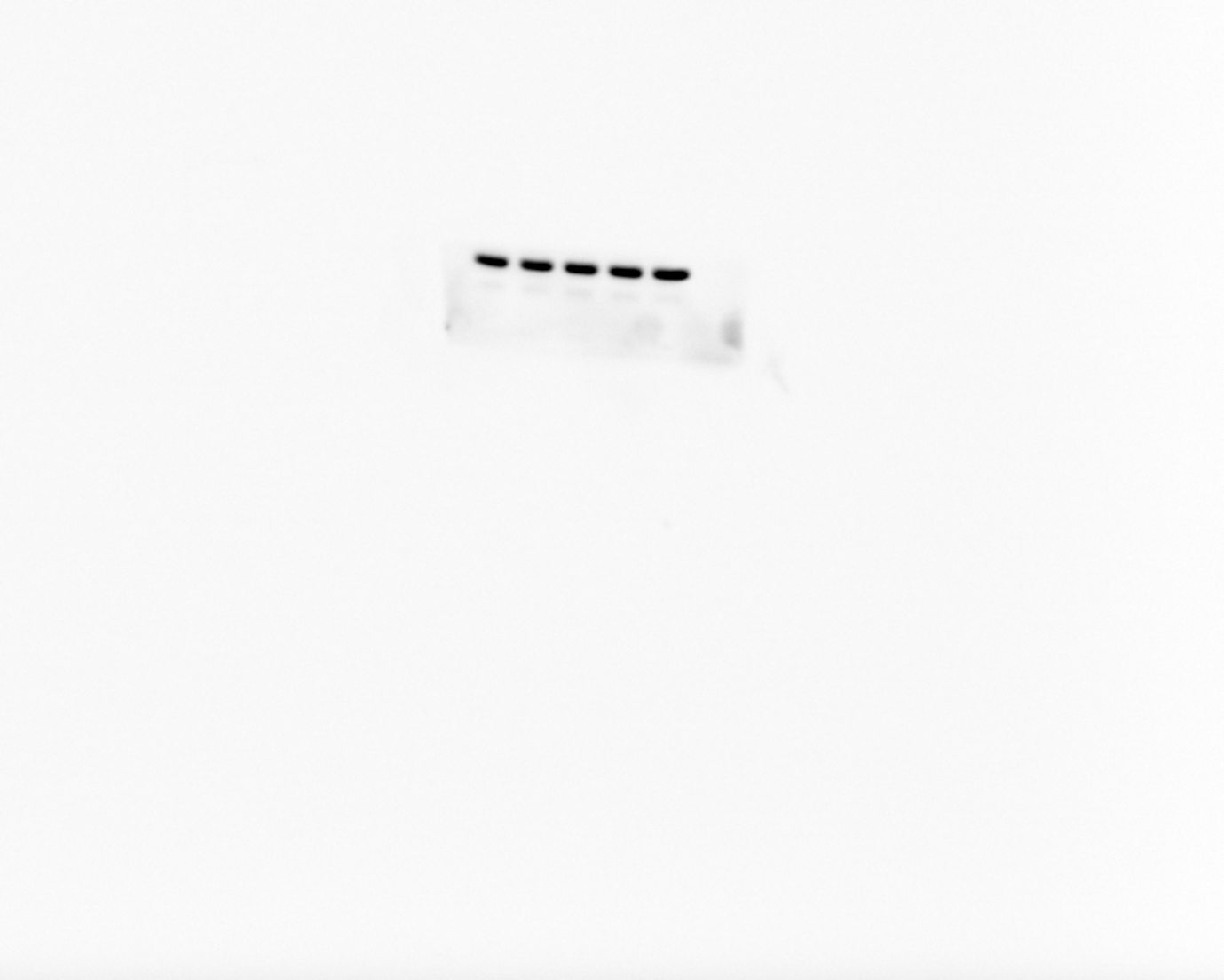

Supplement: Supplementary file 2 [file DataSheet1.ZIP › original blot images-Fig5-9/Fig5B-╬▓-tublin.tif]

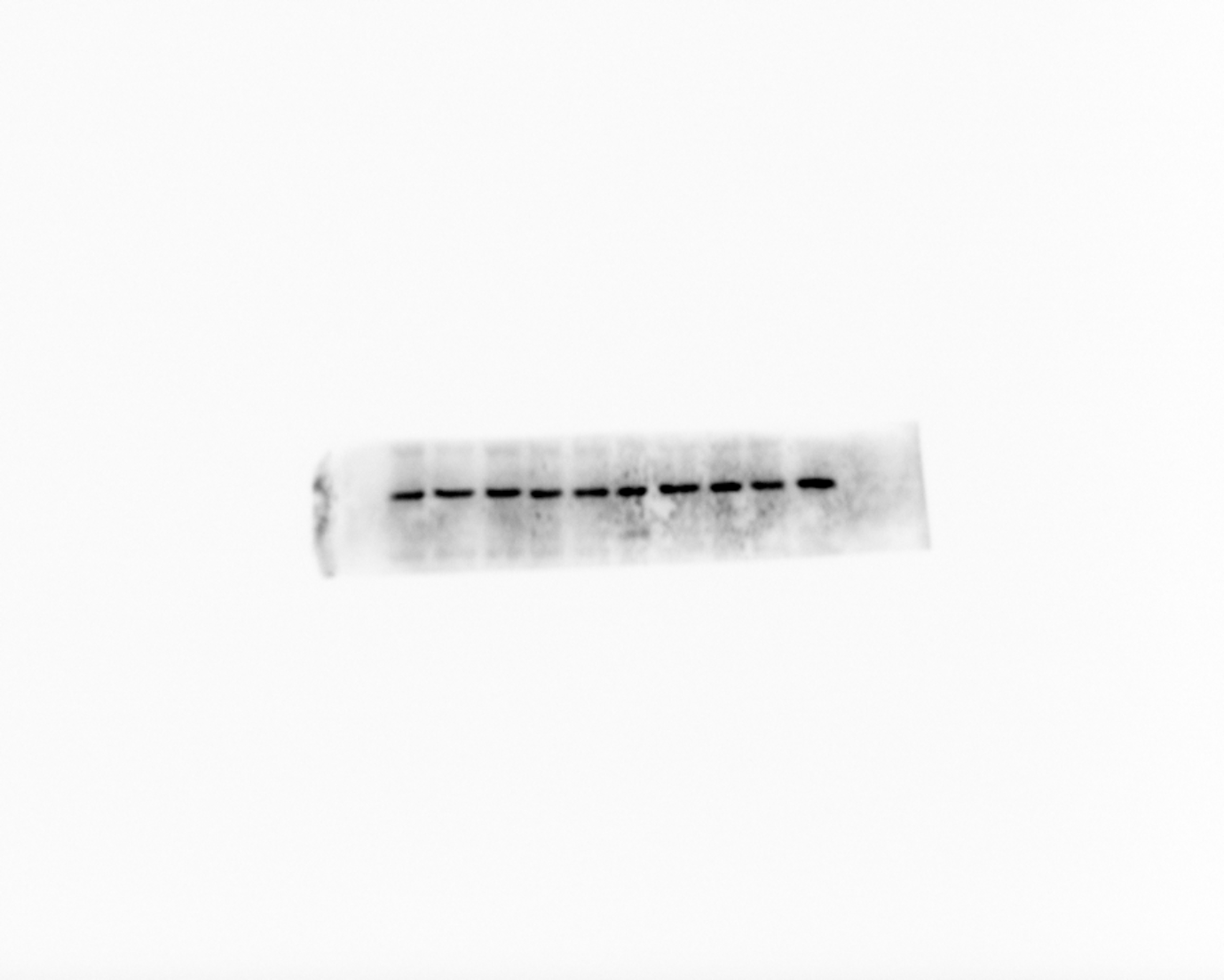

Supplement: Supplementary file 2 [file DataSheet1.ZIP › original blot images-Fig5-9/Fig9D-GAPDH.tif]

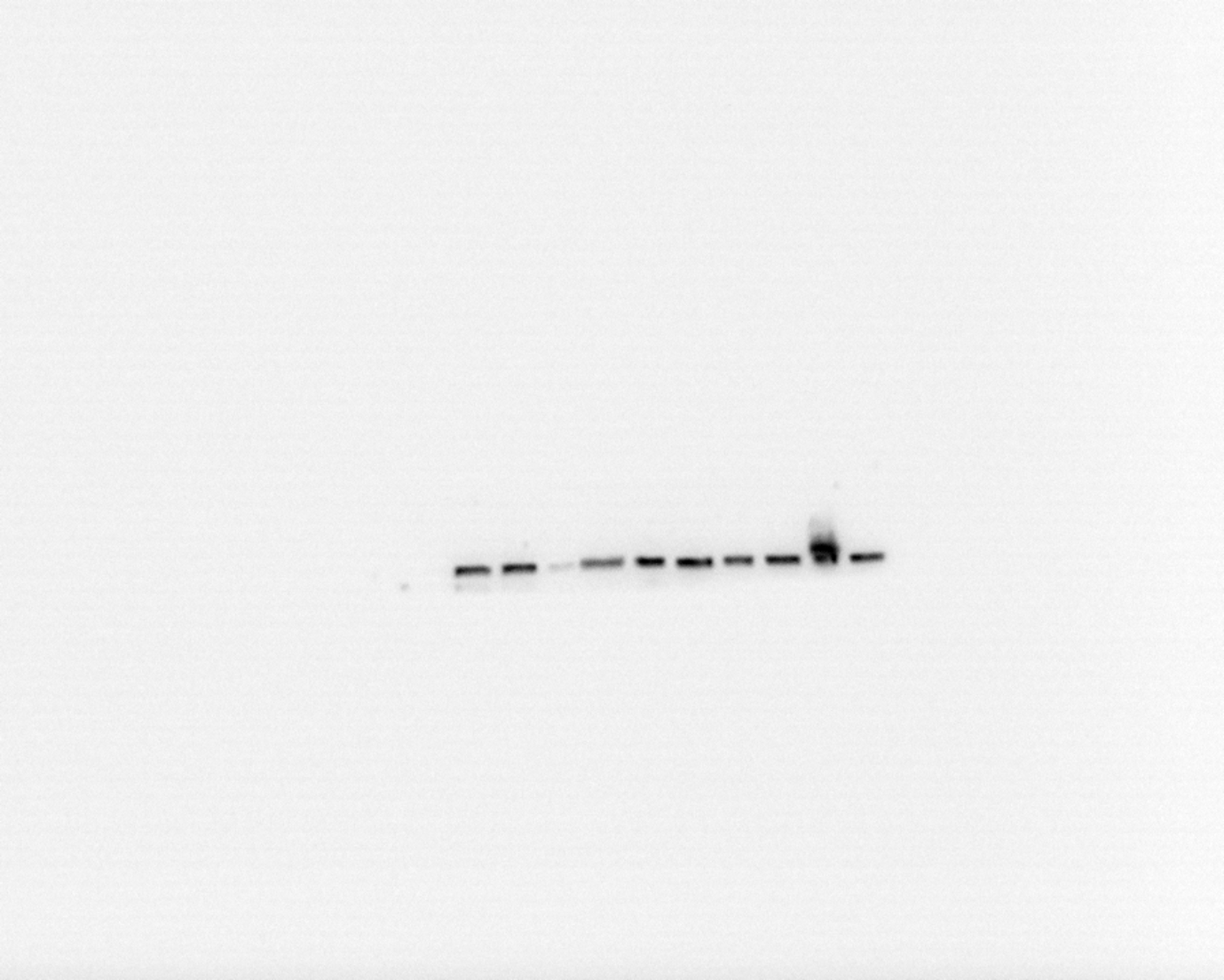

Supplement: Supplementary file 2 [file DataSheet1.ZIP › original blot images-Fig5-9/Fig9D-E-cadherin.tif]

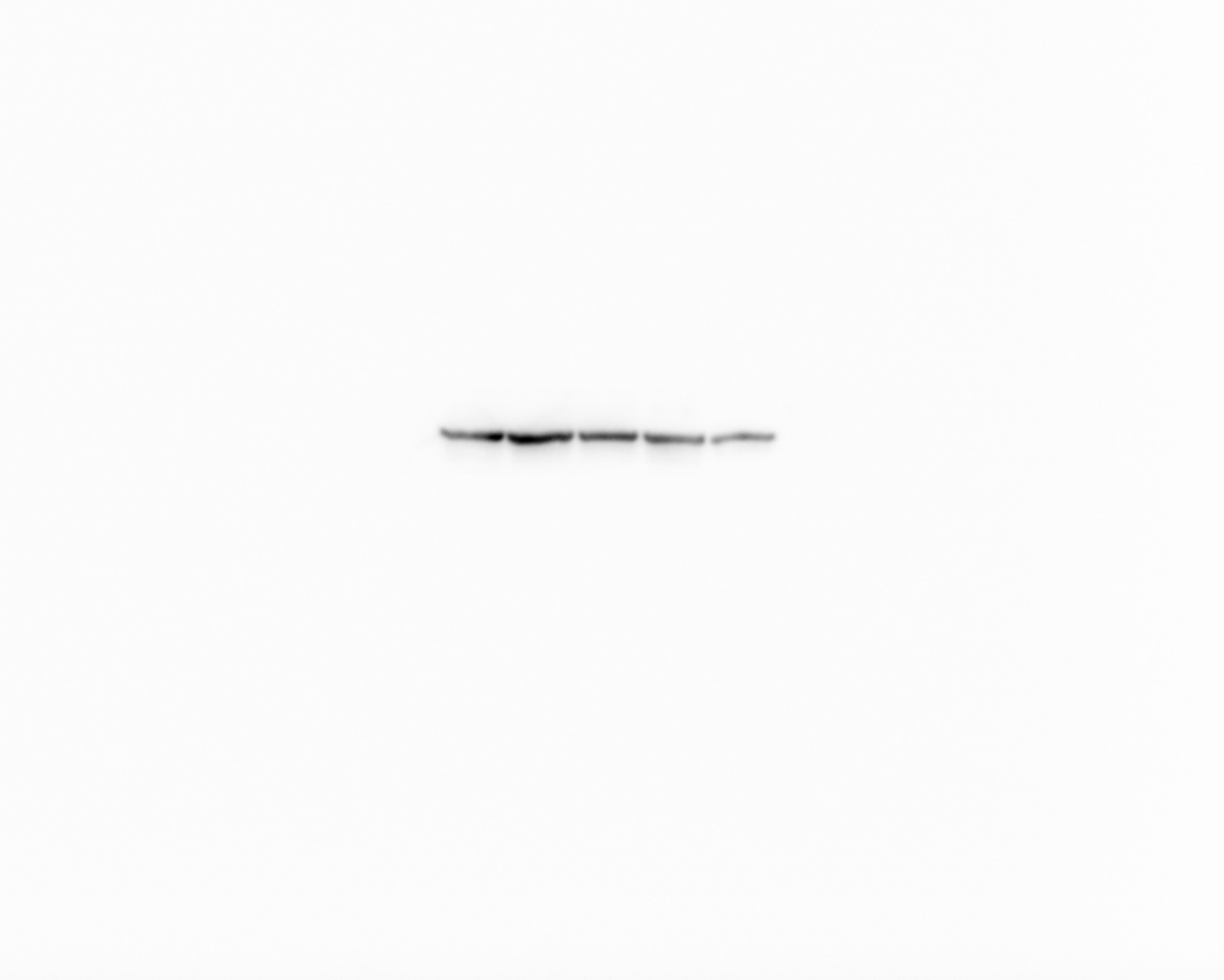

Supplement: Supplementary file 2 [file DataSheet1.ZIP › original blot images-Fig5-9/Fig7C-Vimentin.tif]

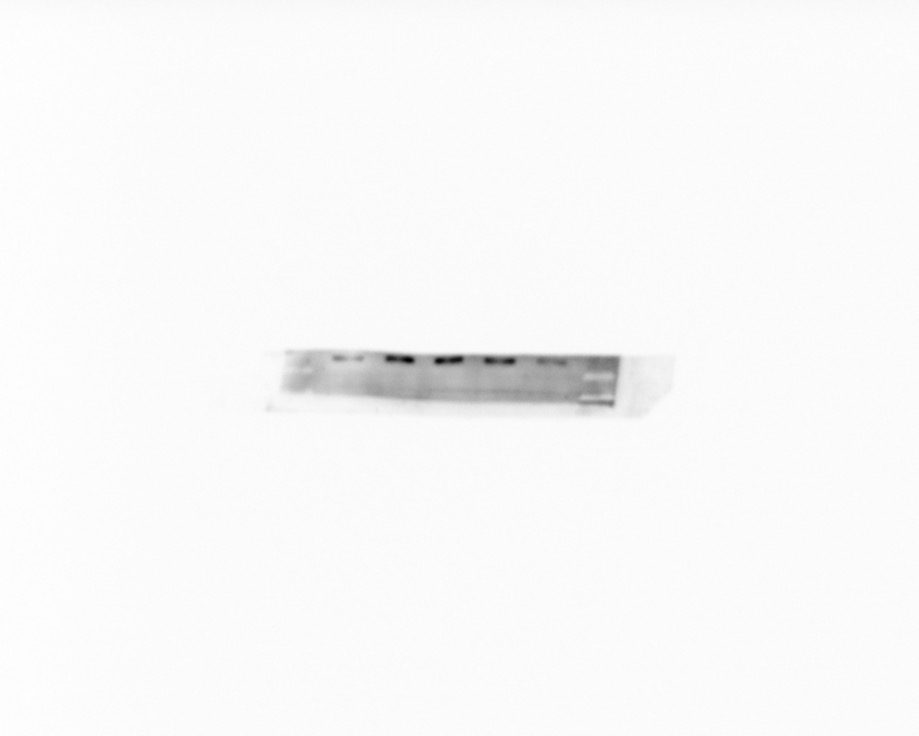

Supplement: Supplementary file 2 [file DataSheet1.ZIP › original blot images-Fig5-9/Fig5B-SMA.jpg]

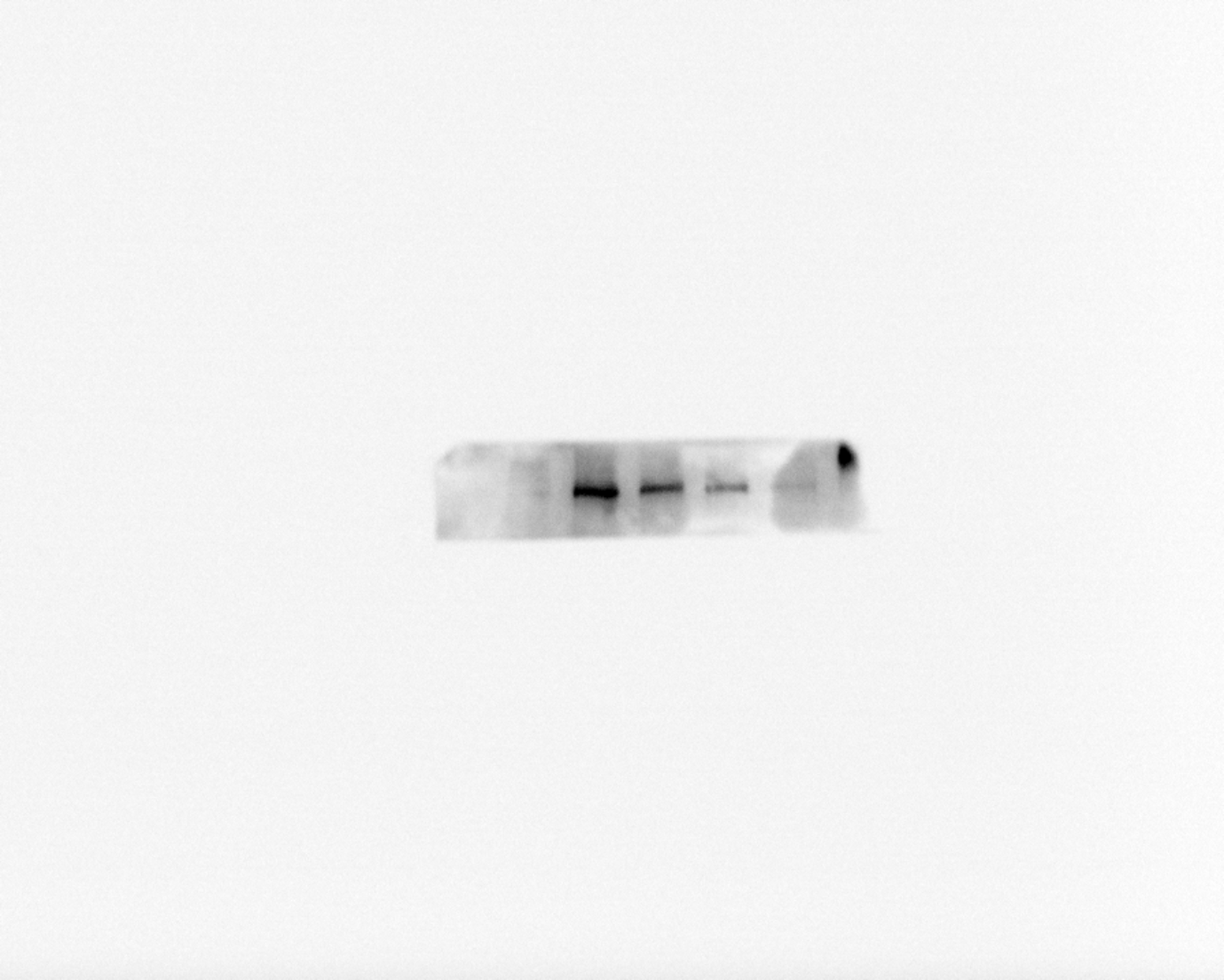

Supplement: Supplementary file 2 [file DataSheet1.ZIP › original blot images-Fig5-9/Fig5C-Fn.tif]

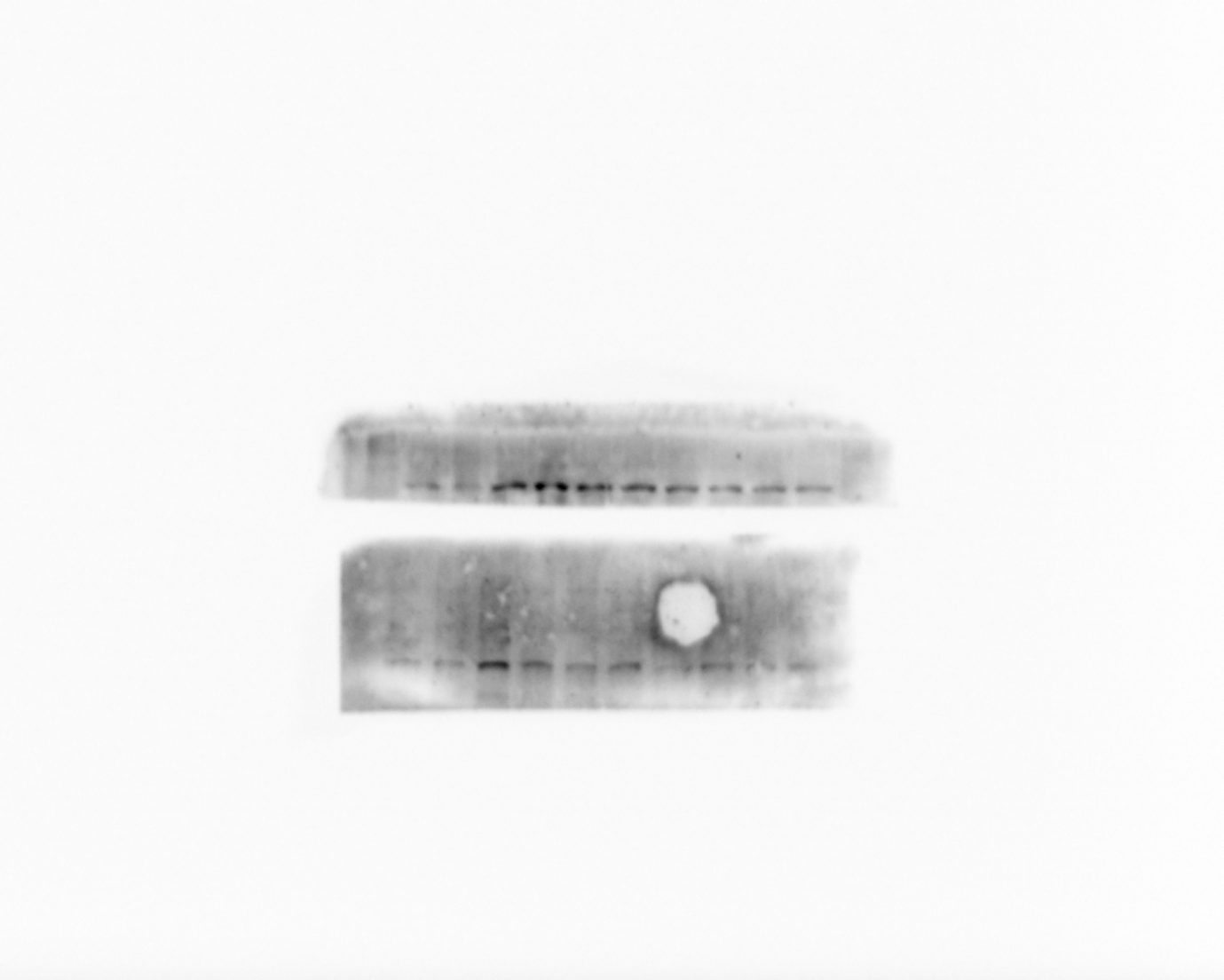

Supplement: Supplementary file 2 [file DataSheet1.ZIP › original blot images-Fig5-9/Fig9B-Col-1.tif]

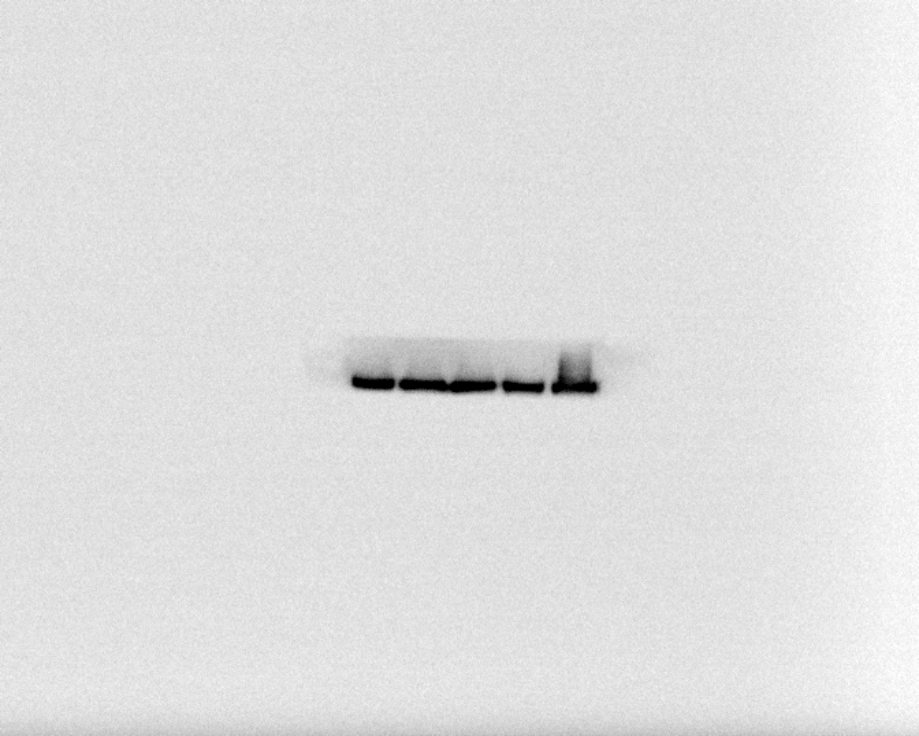

Supplement: Supplementary file 2 [file DataSheet1.ZIP › original blot images-Fig5-9/Fig6C-Smad3.tif]

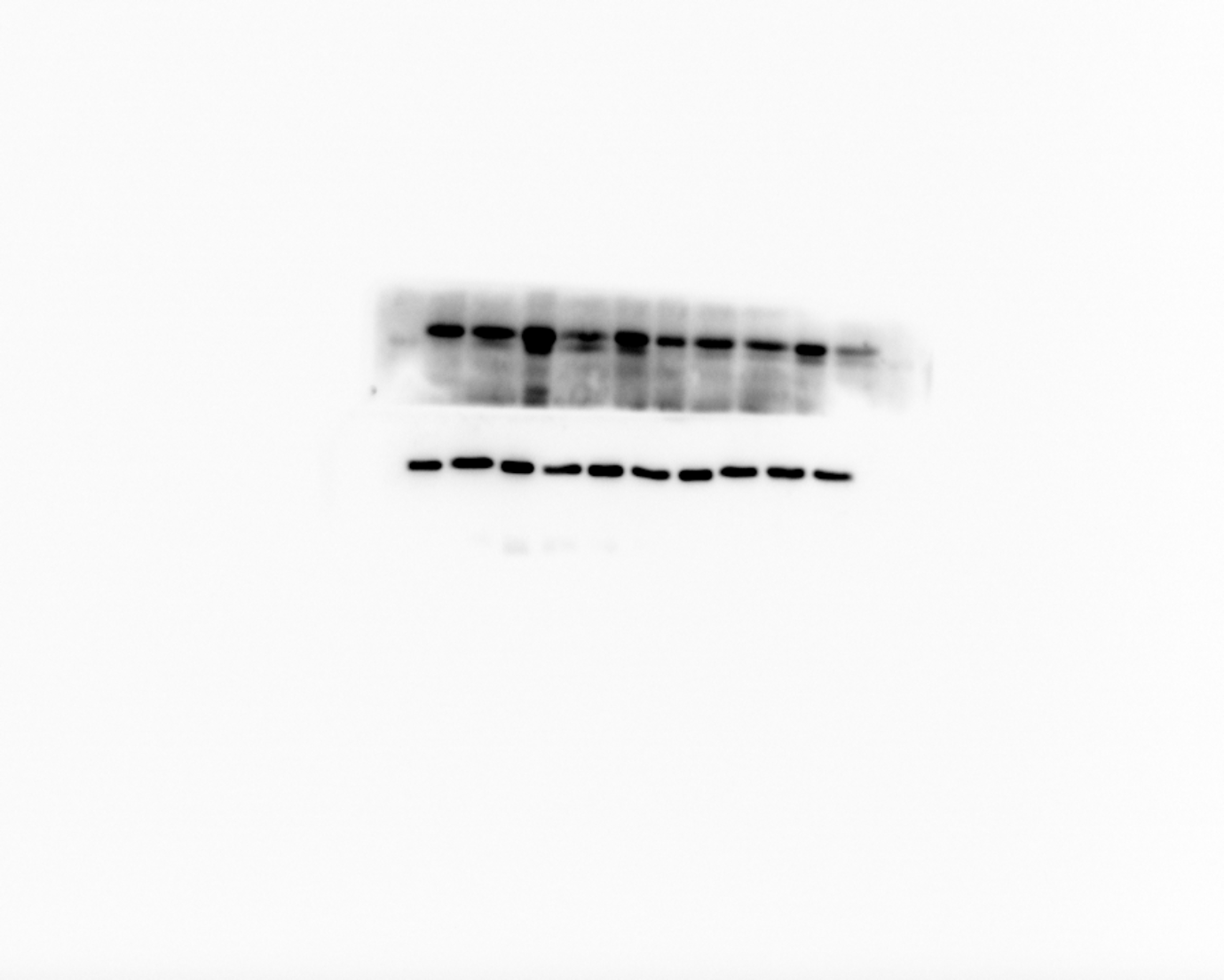

Supplement: Supplementary file 2 [file DataSheet1.ZIP › original blot images-Fig5-9/Fig9B-GAPDH.tif]

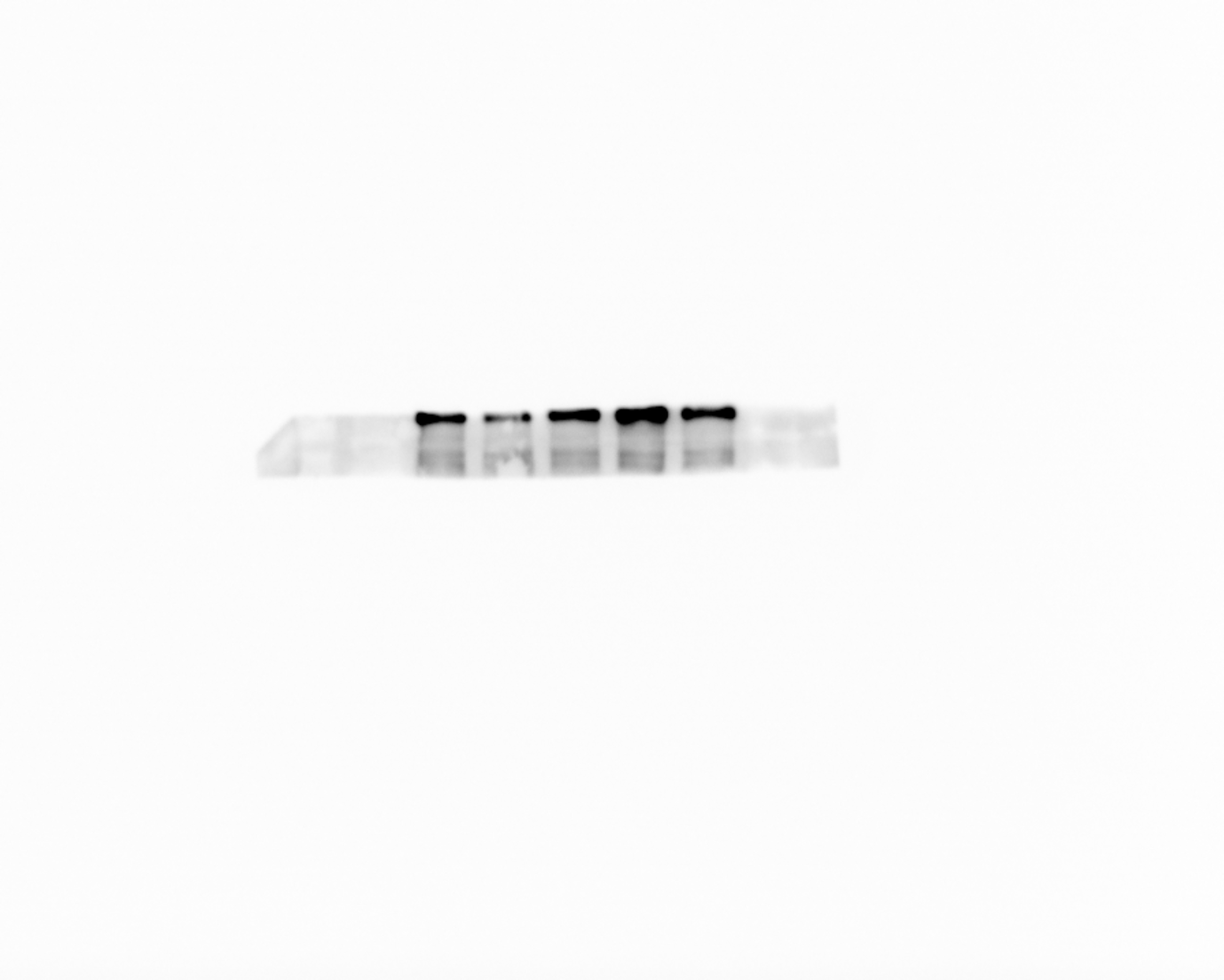

Supplement: Supplementary file 2 [file DataSheet1.ZIP › original blot images-Fig5-9/Fig7C-E-Cad.tif]

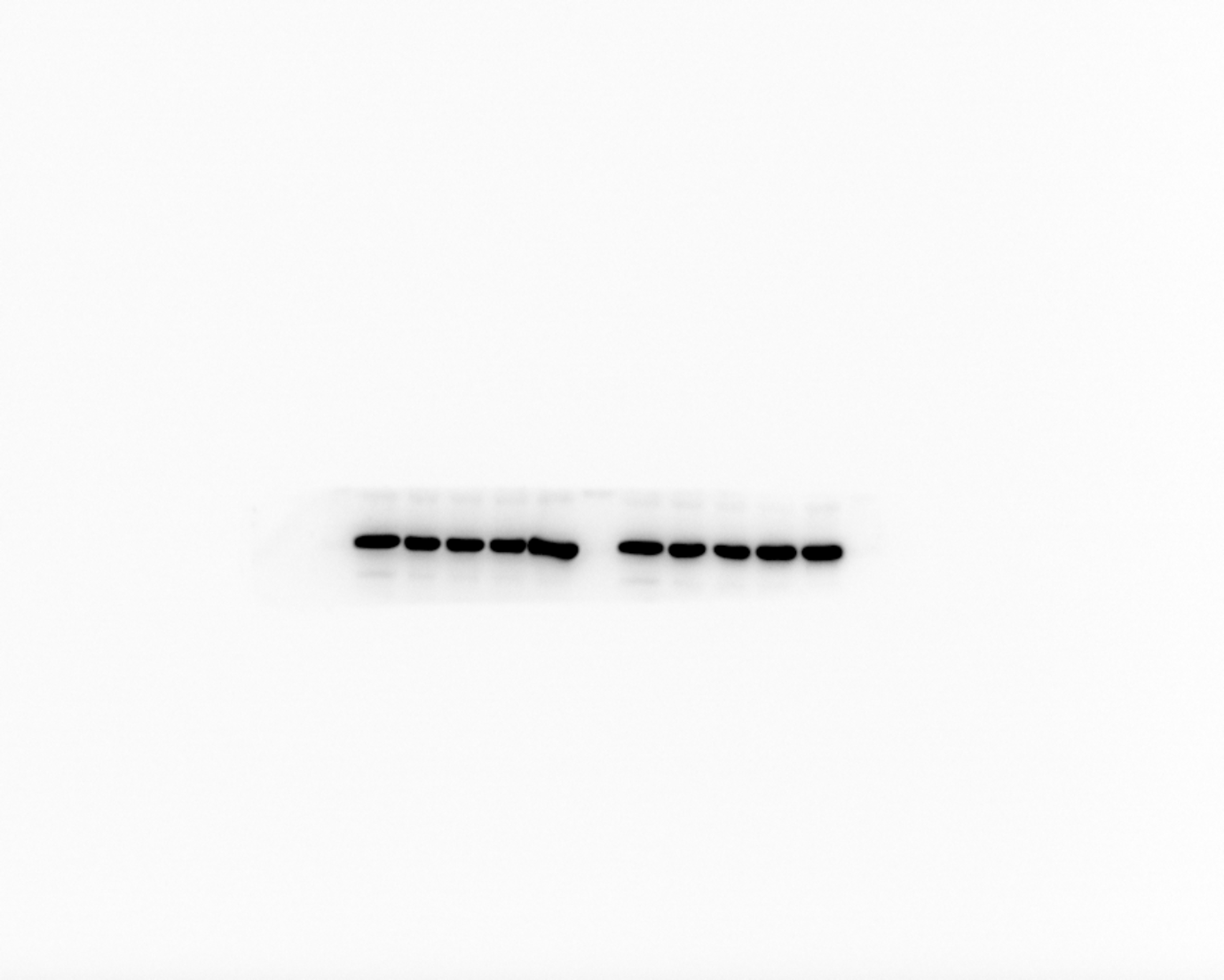

Supplement: Supplementary file 2 [file DataSheet1.ZIP › original blot images-Fig5-9/Fig6c-GAPDH.tif]

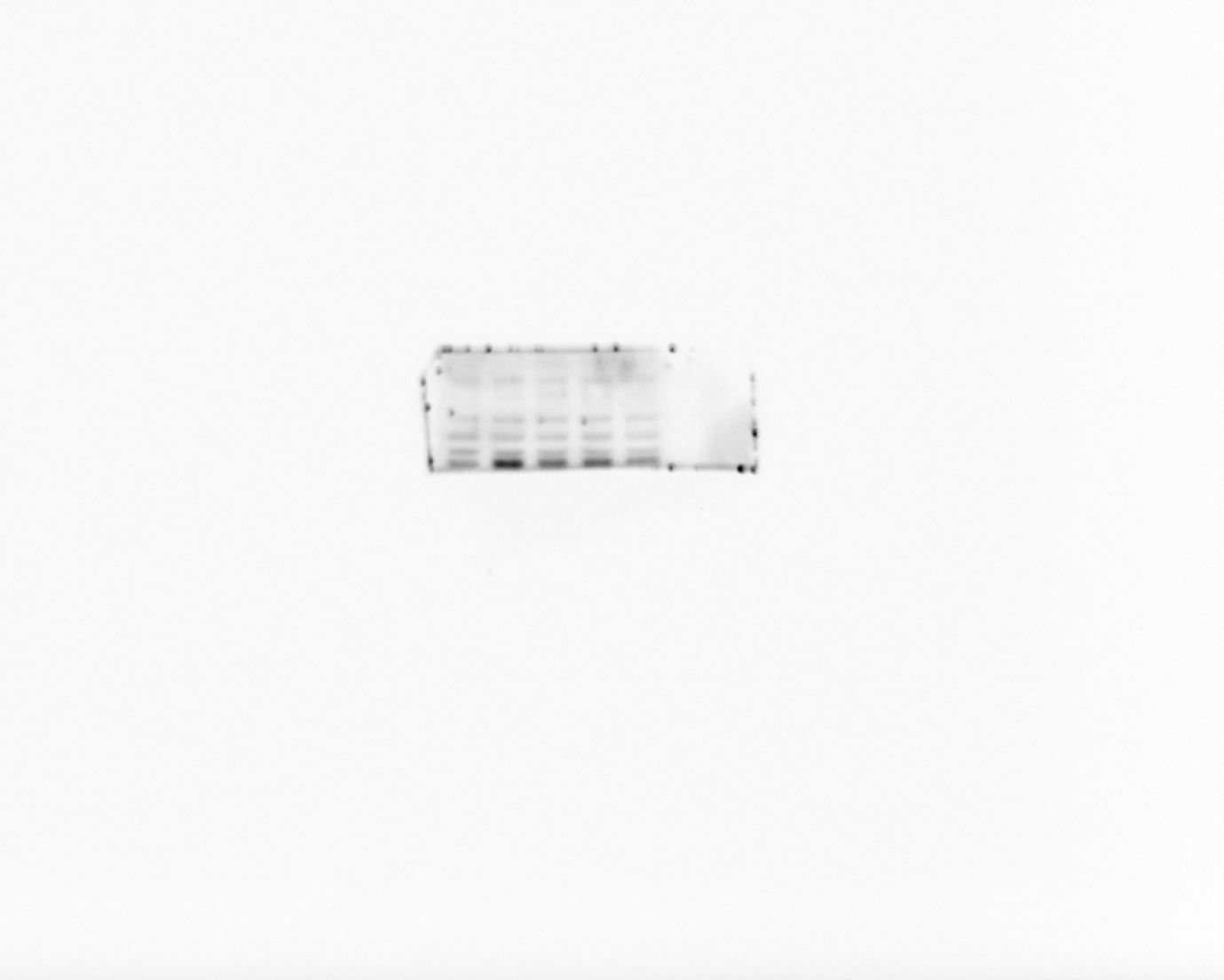

Supplement: Supplementary file 2 [file DataSheet1.ZIP › original blot images-Fig5-9/Fig6D-p-p38.tif]

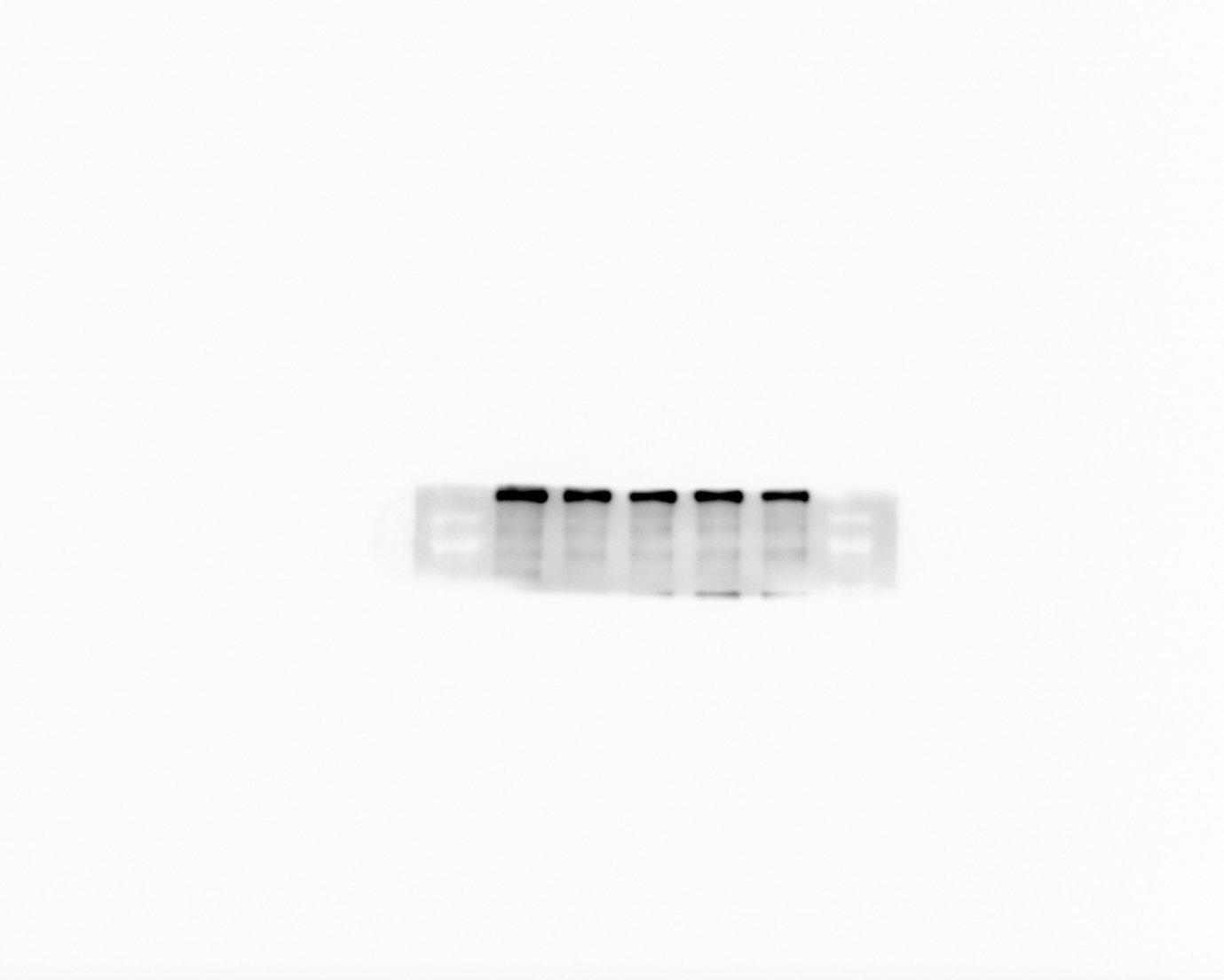

Supplement: Supplementary file 2 [file DataSheet1.ZIP › original blot images-Fig5-9/Fig6D-JNK.tif]

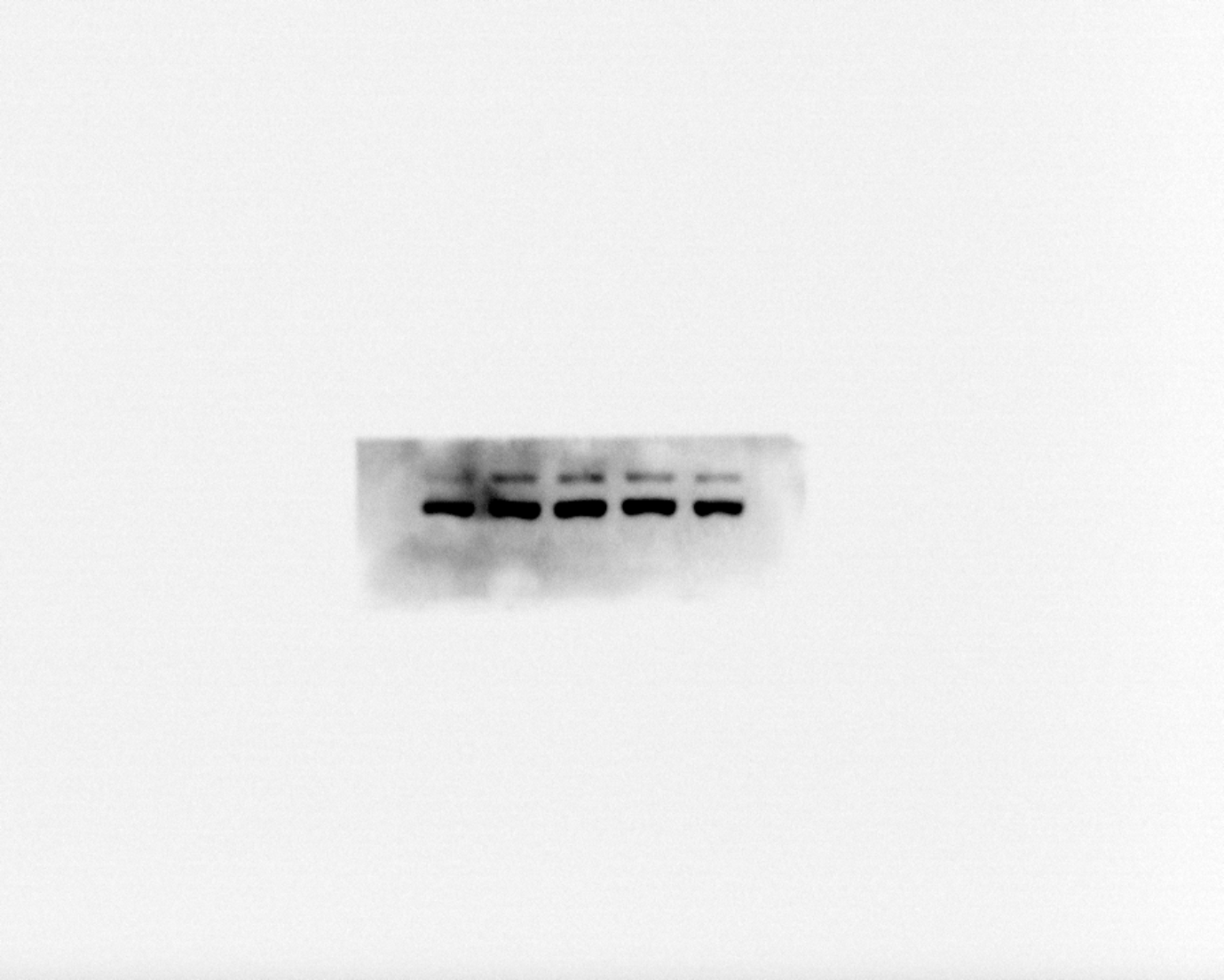

Supplement: Supplementary file 2 [file DataSheet1.ZIP › original blot images-Fig5-9/Fig6D-ERK.tif]

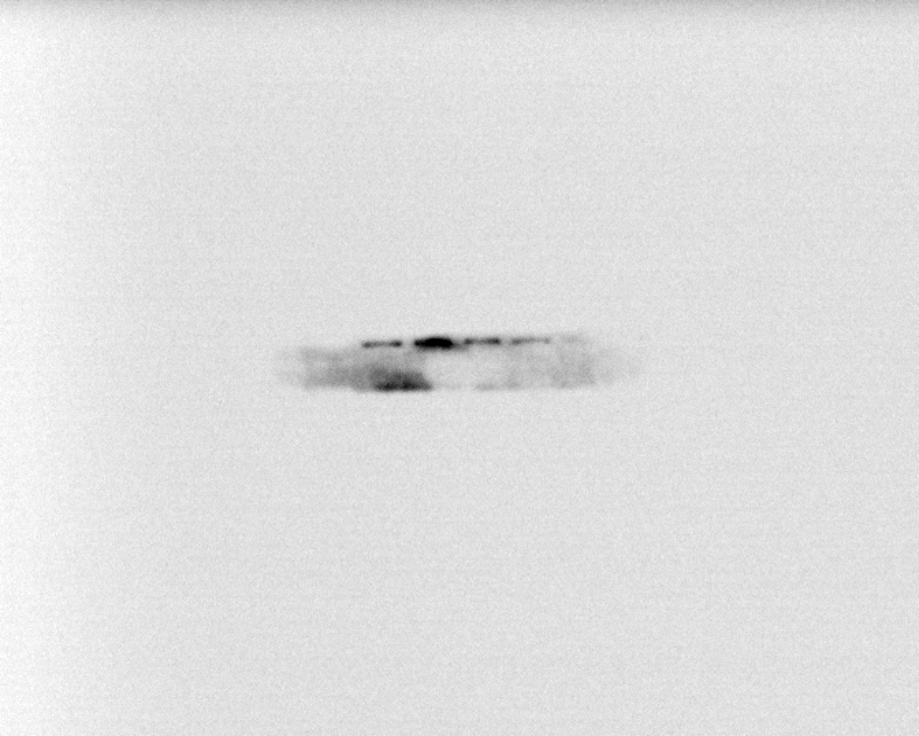

Supplement: Supplementary file 2 [file DataSheet1.ZIP › original blot images-Fig5-9/Fig6B-p-Smad3.tif]

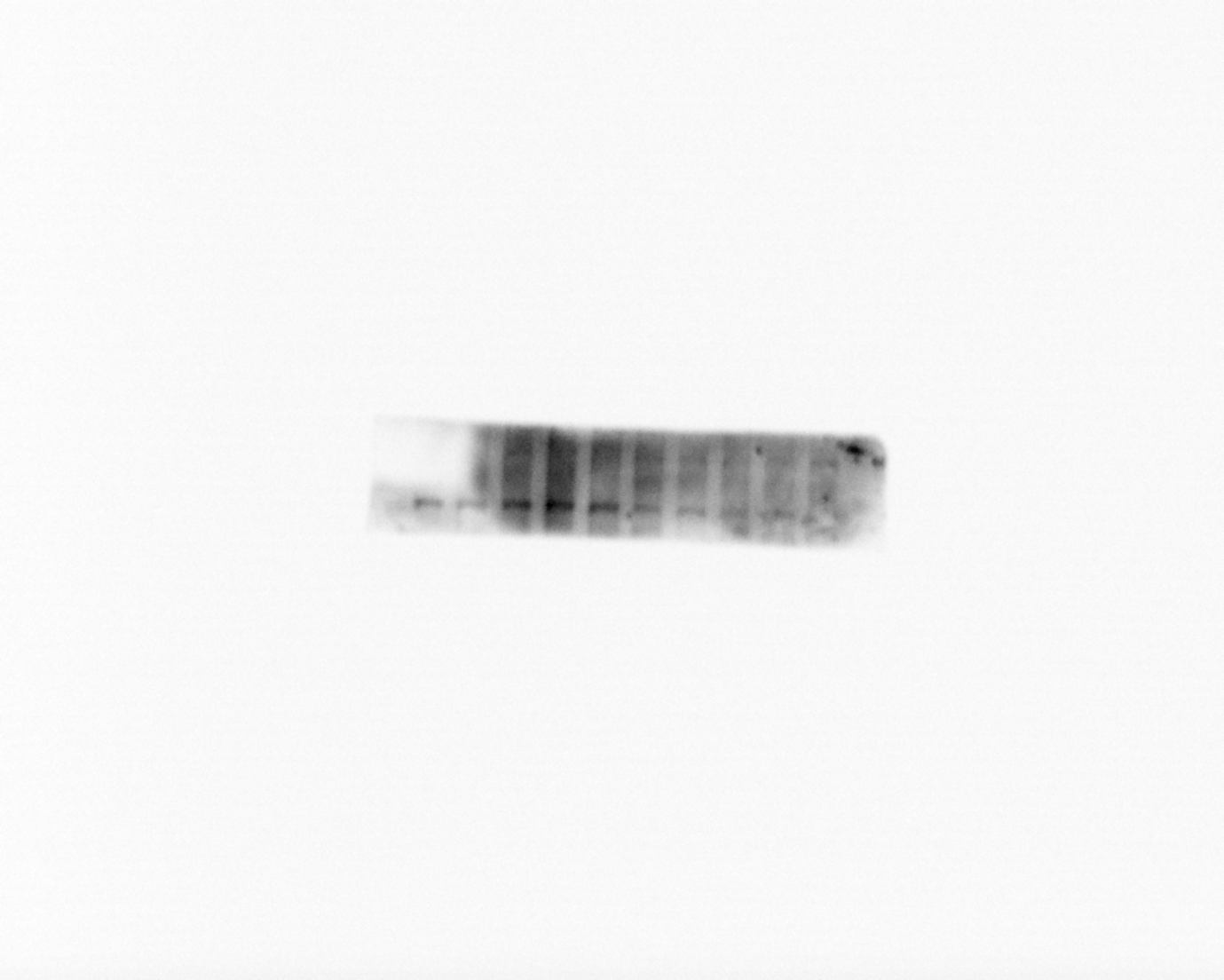

Supplement: Supplementary file 2 [file DataSheet1.ZIP › original blot images-Fig5-9/Fig9D-N-Cadherin.tif]

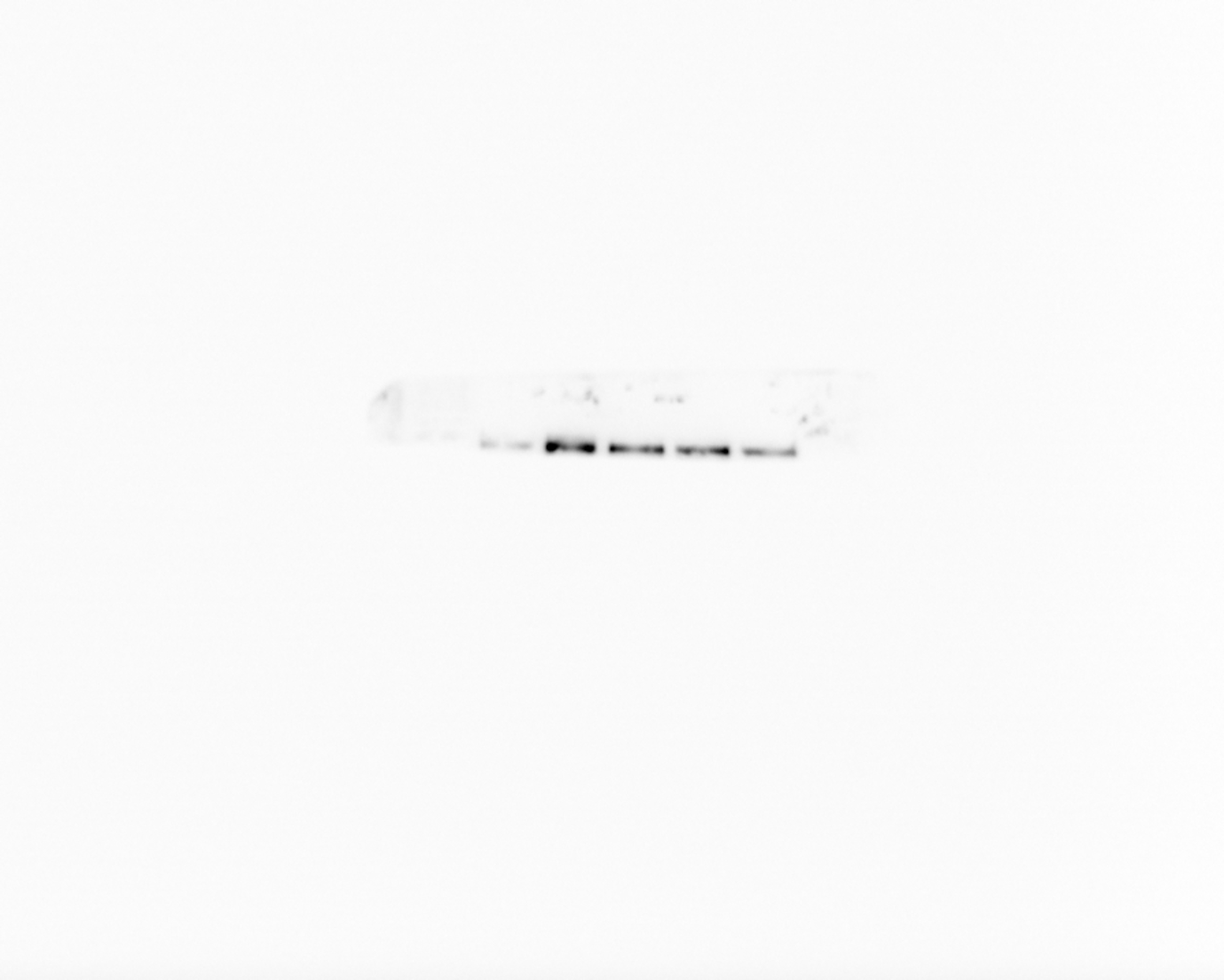

Supplement: Supplementary file 2 [file DataSheet1.ZIP › original blot images-Fig5-9/Fig7C-N-Cad.tif]

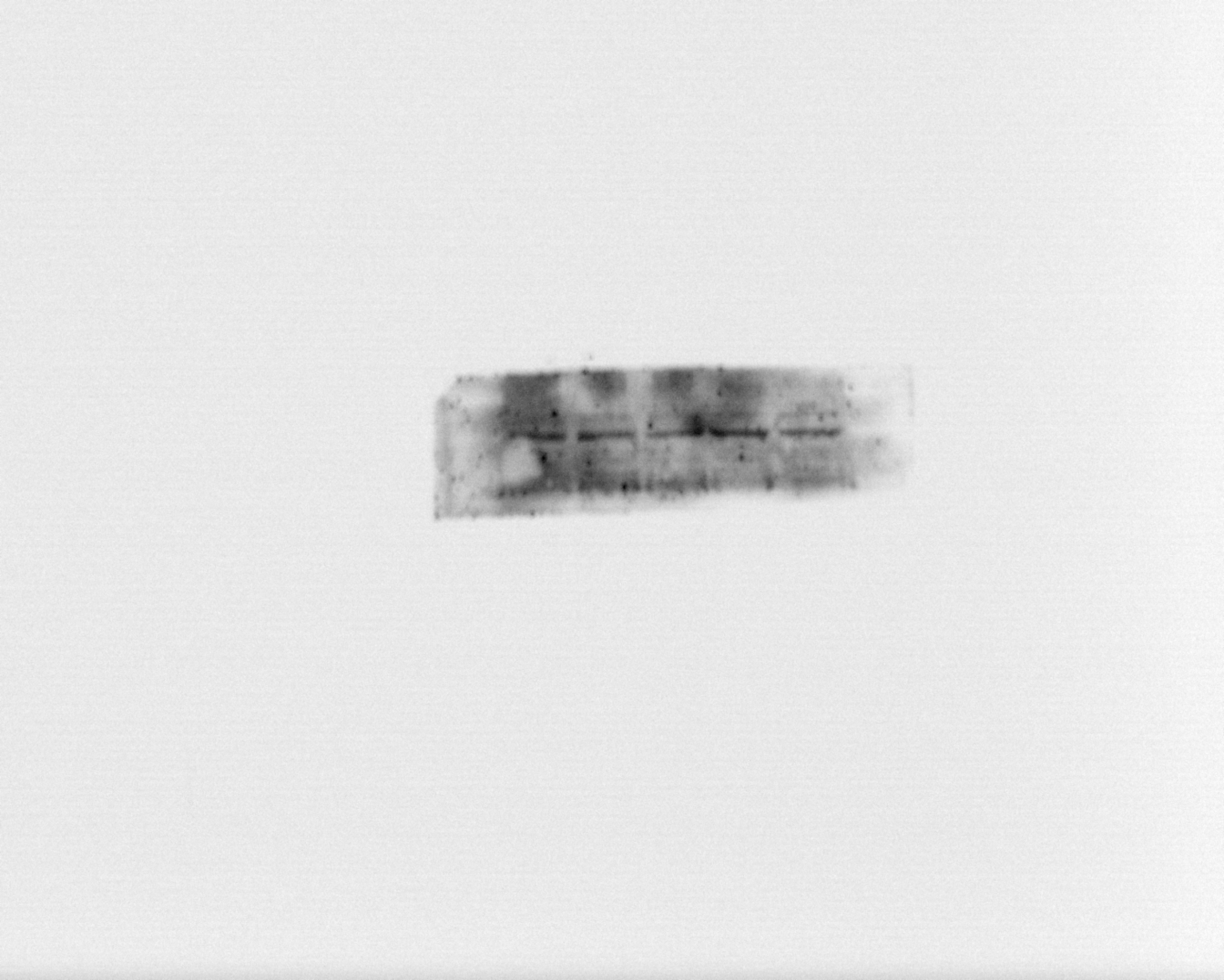

Supplement: Supplementary file 2 [file DataSheet1.ZIP › original blot images-Fig5-9/Fig6B-Smad3.tif]

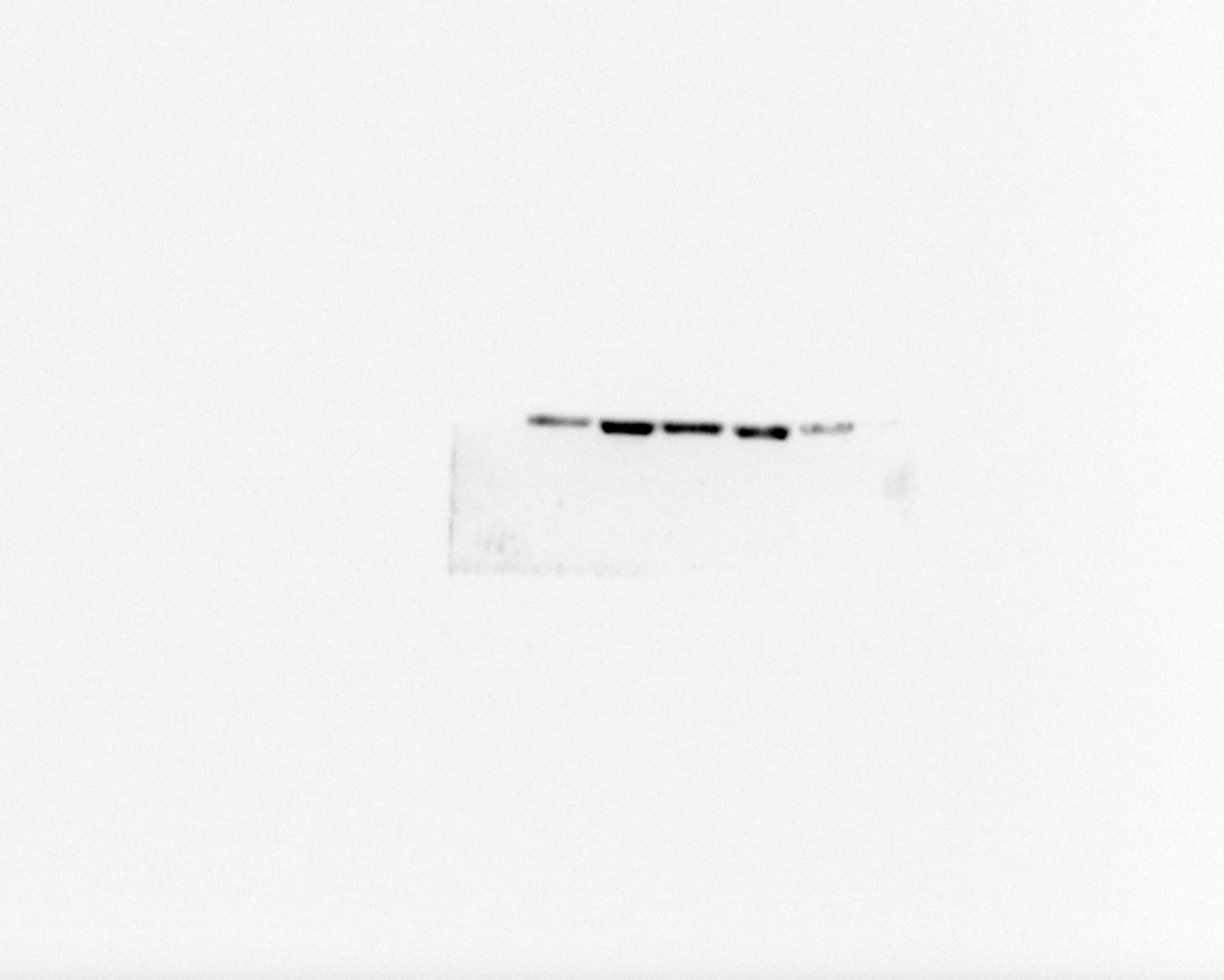

Supplement: Supplementary file 2 [file DataSheet1.ZIP › original blot images-Fig5-9/Fig5C-╬▒-SMA.tif]

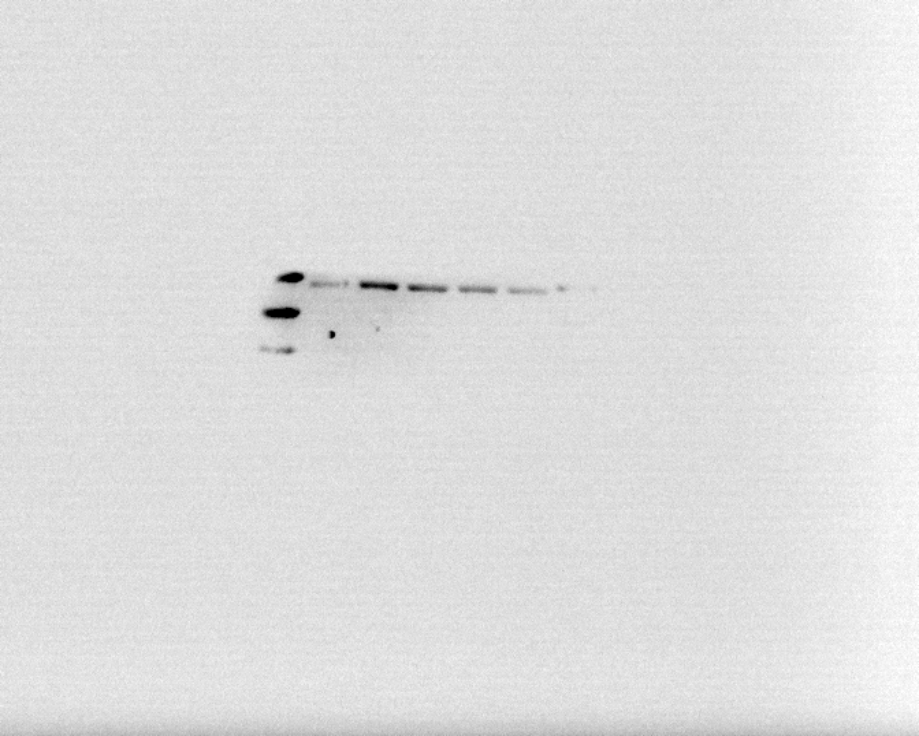

Supplement: Supplementary file 2 [file DataSheet1.ZIP › original blot images-Fig5-9/Fig6D-P-JNK.tif]

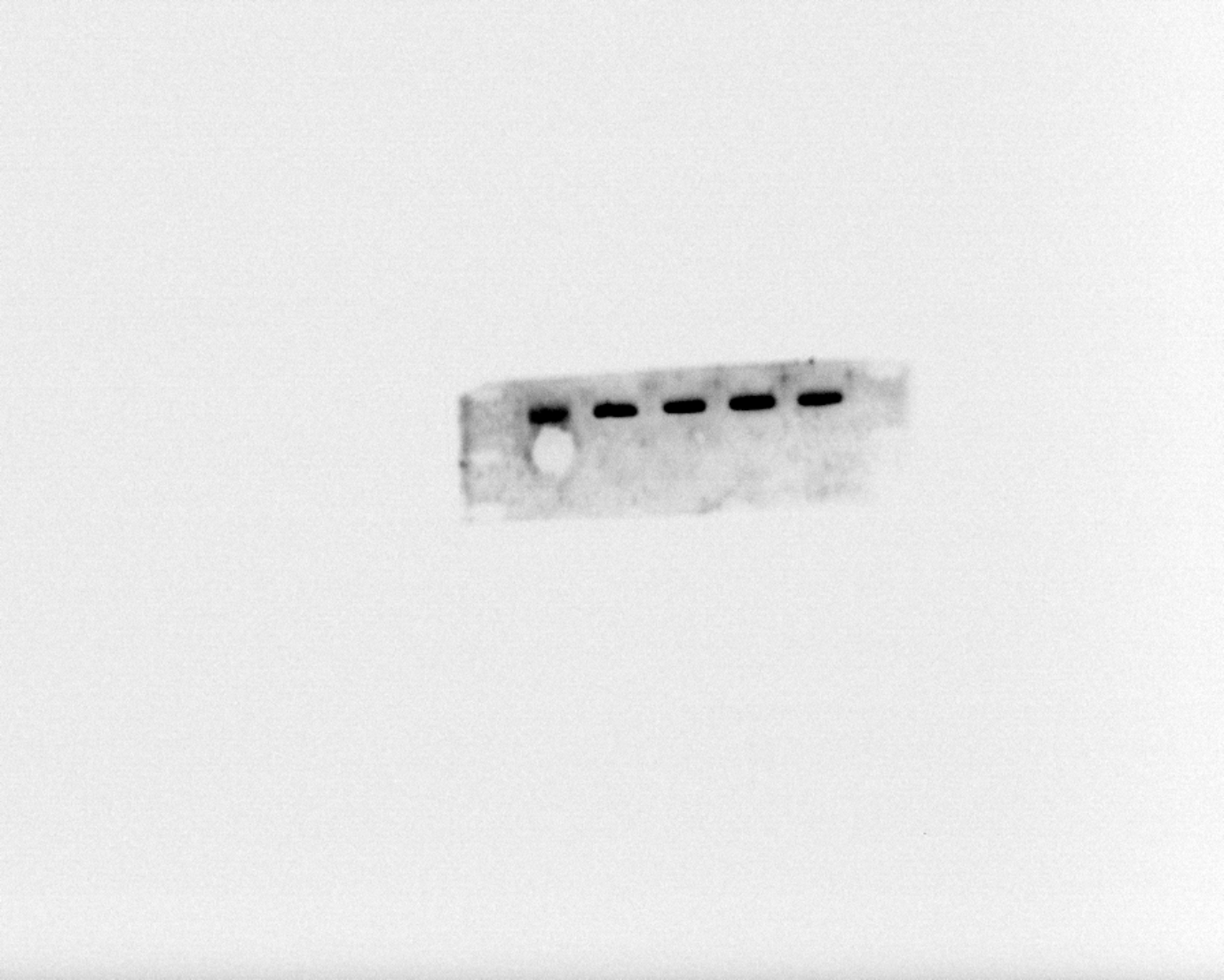

Supplement: Supplementary file 2 [file DataSheet1.ZIP › original blot images-Fig5-9/Fig6D-P38.tif]

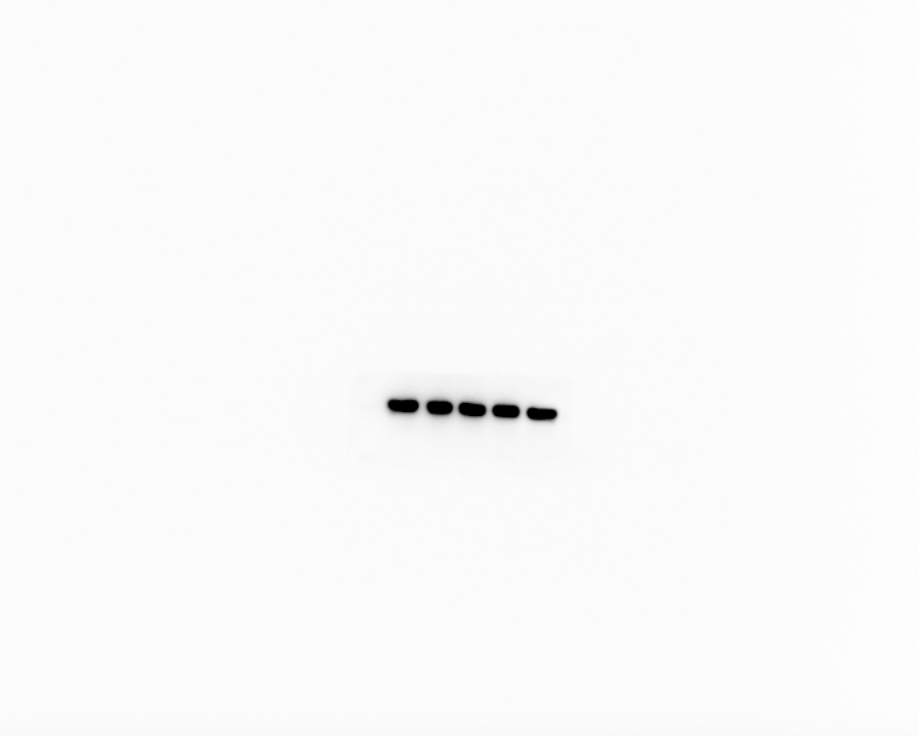

Supplement: Supplementary file 2 [file DataSheet1.ZIP › original blot images-Fig5-9/Fig6B-GAPDH.tif]

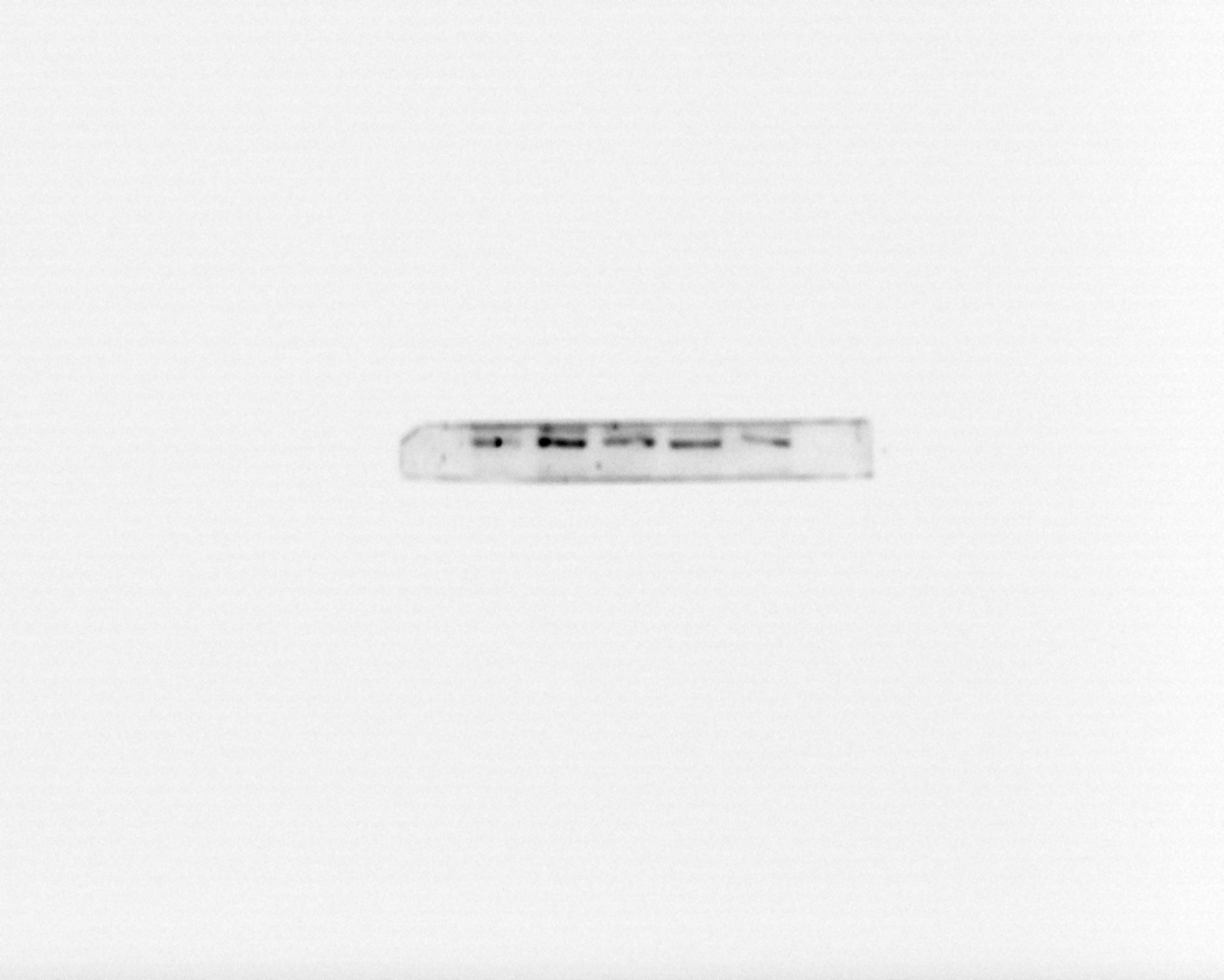

Supplement: Supplementary file 2 [file DataSheet1.ZIP › original blot images-Fig5-9/Fig6D-p-ERK.tif]

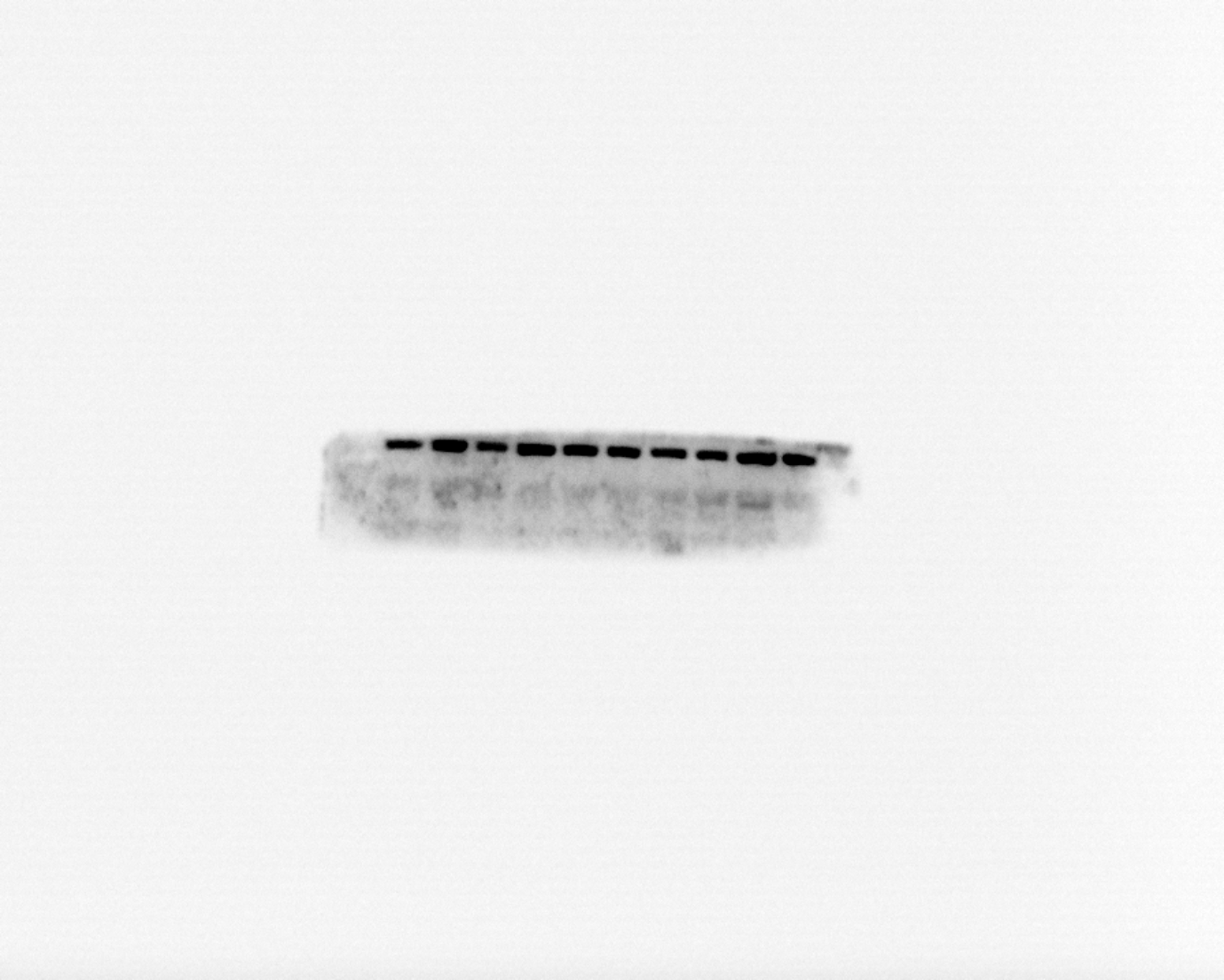

Supplement: Supplementary file 3 [file DataSheet2.ZIP › original blot images-Fig10/Fig10-smad2.tif]

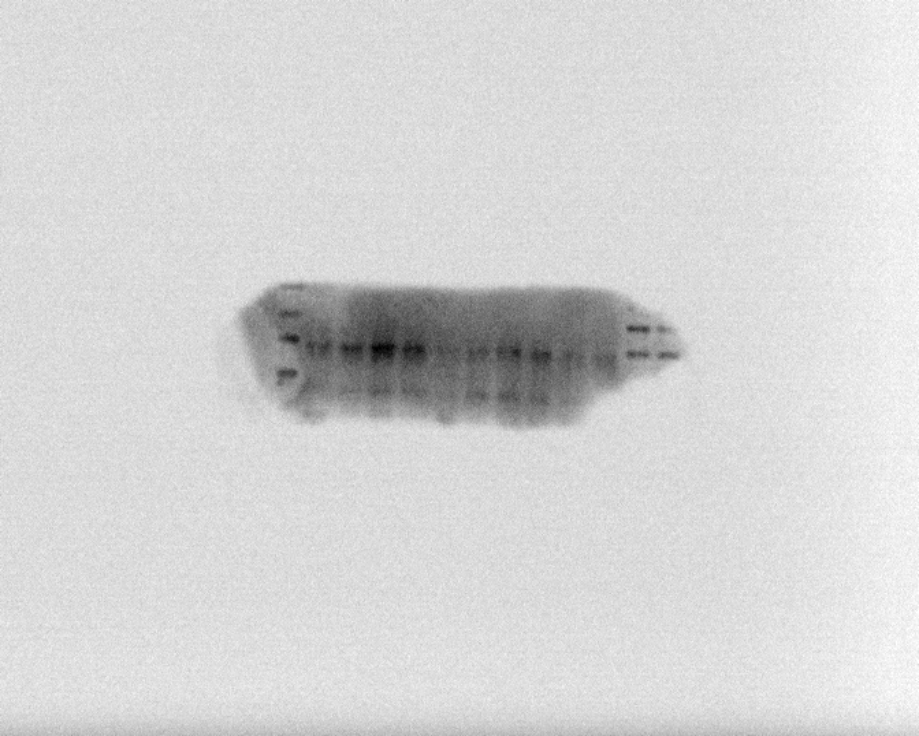

Supplement: Supplementary file 3 [file DataSheet2.ZIP › original blot images-Fig10/Fig10-p-p38.tif]

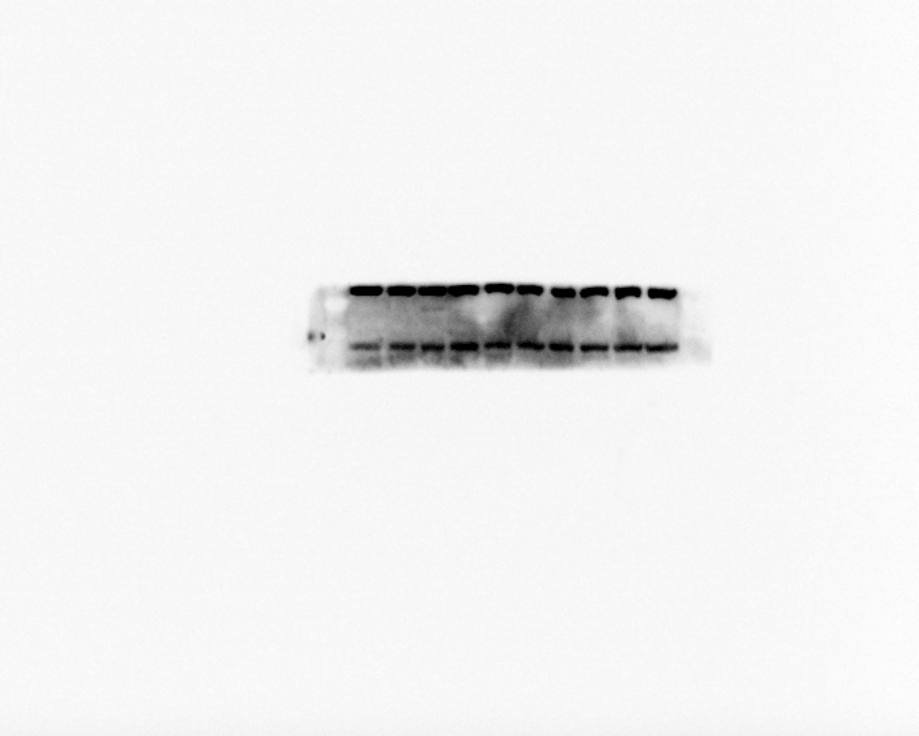

Supplement: Supplementary file 3 [file DataSheet2.ZIP › original blot images-Fig10/Fig10-Smad3.tif]

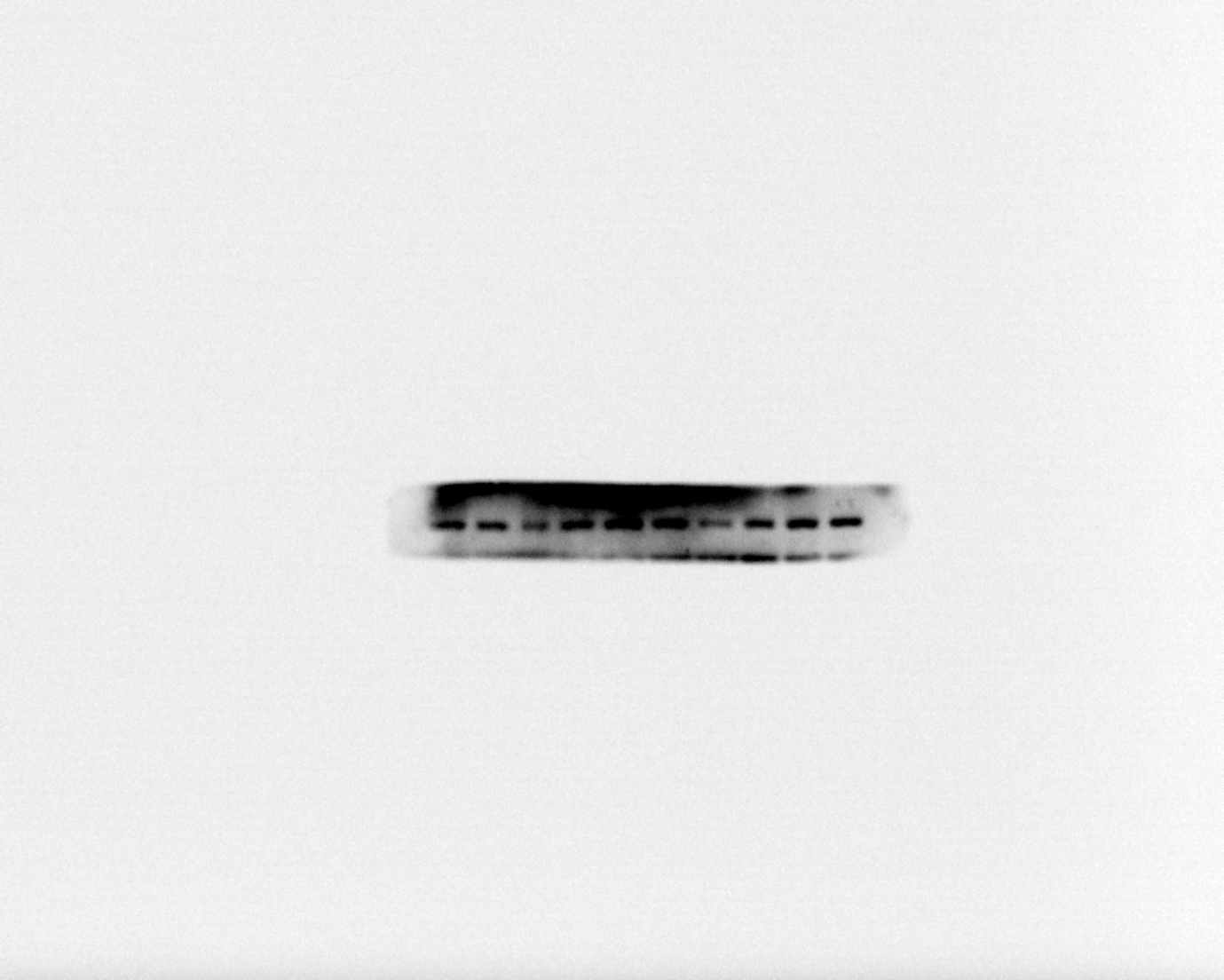

Supplement: Supplementary file 3 [file DataSheet2.ZIP › original blot images-Fig10/Fig10-p38.tif]

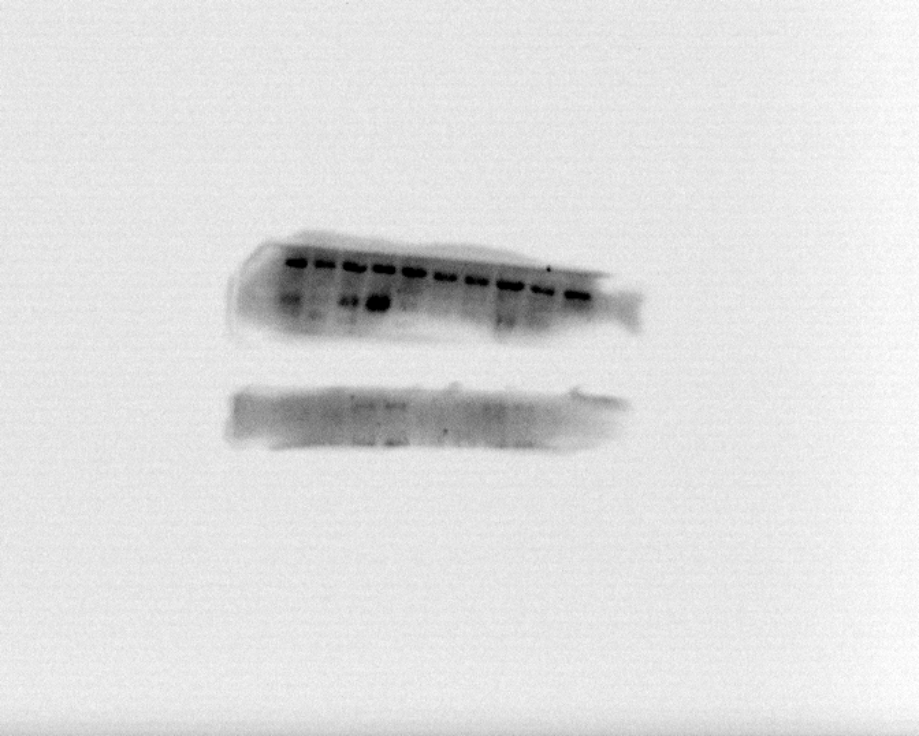

Supplement: Supplementary file 3 [file DataSheet2.ZIP › original blot images-Fig10/Fig10-GAPDH.tif]

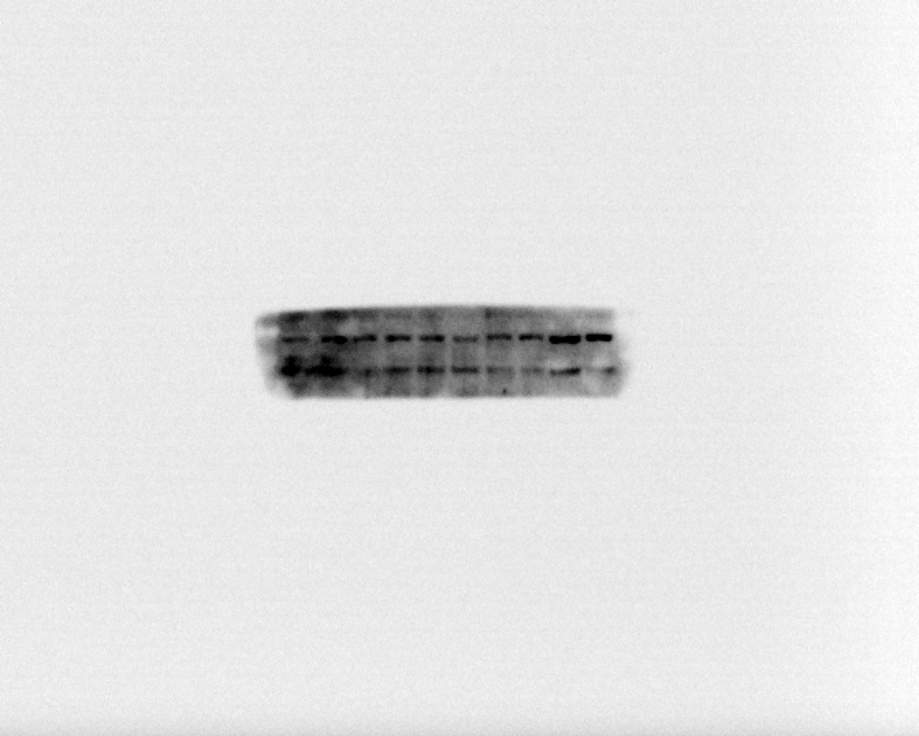

Supplement: Supplementary file 3 [file DataSheet2.ZIP › original blot images-Fig10/Fig10-JNK.tif]

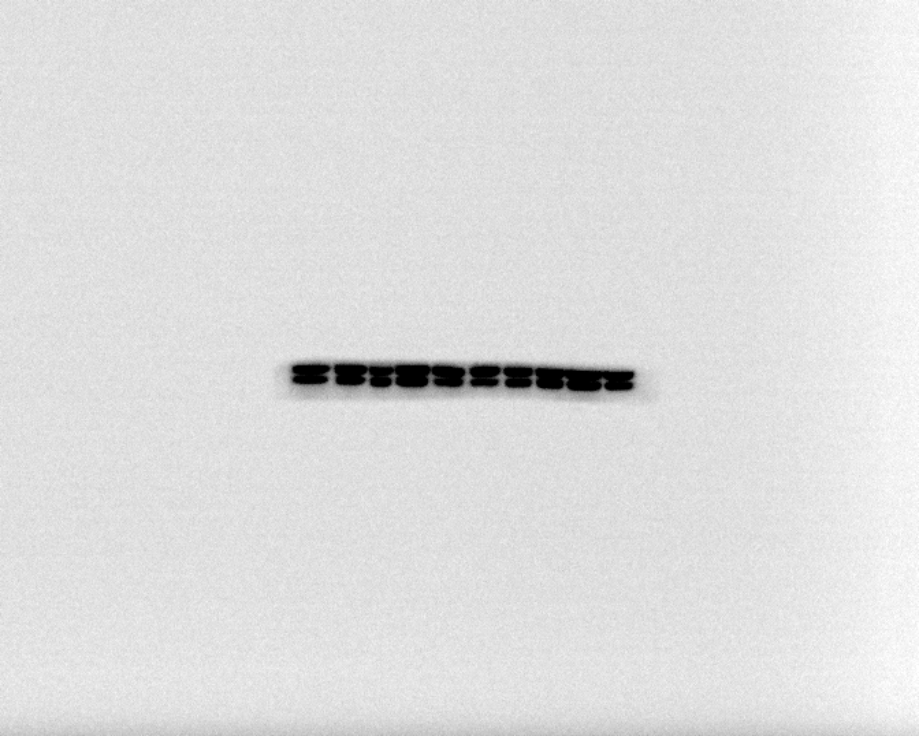

Supplement: Supplementary file 3 [file DataSheet2.ZIP › original blot images-Fig10/Fig10-ERK.tif]

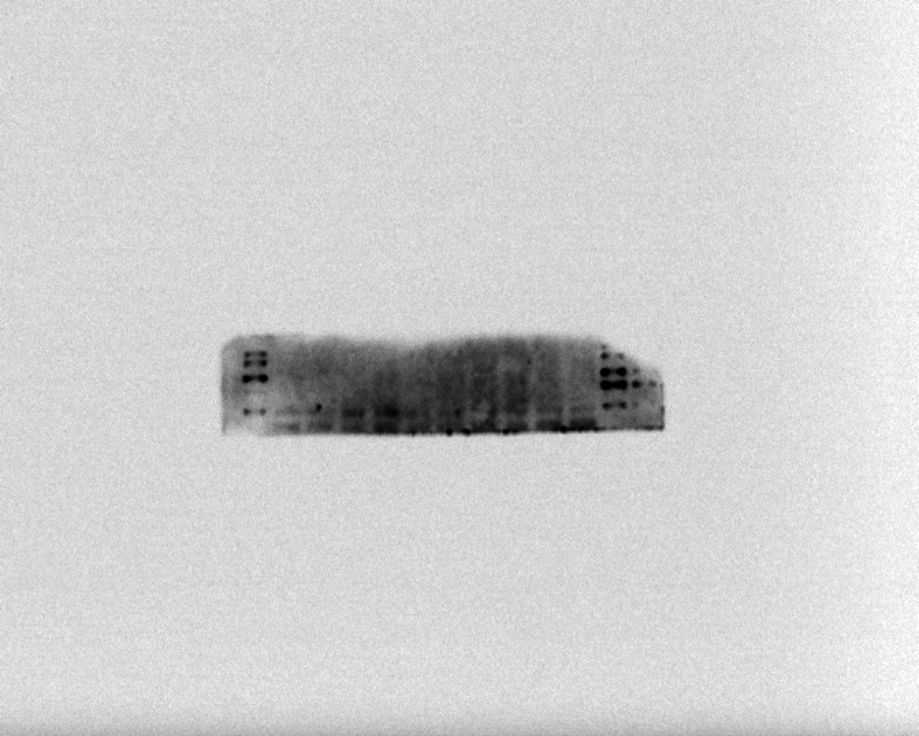

Supplement: Supplementary file 3 [file DataSheet2.ZIP › original blot images-Fig10/Fig10-p-JNK.tif]

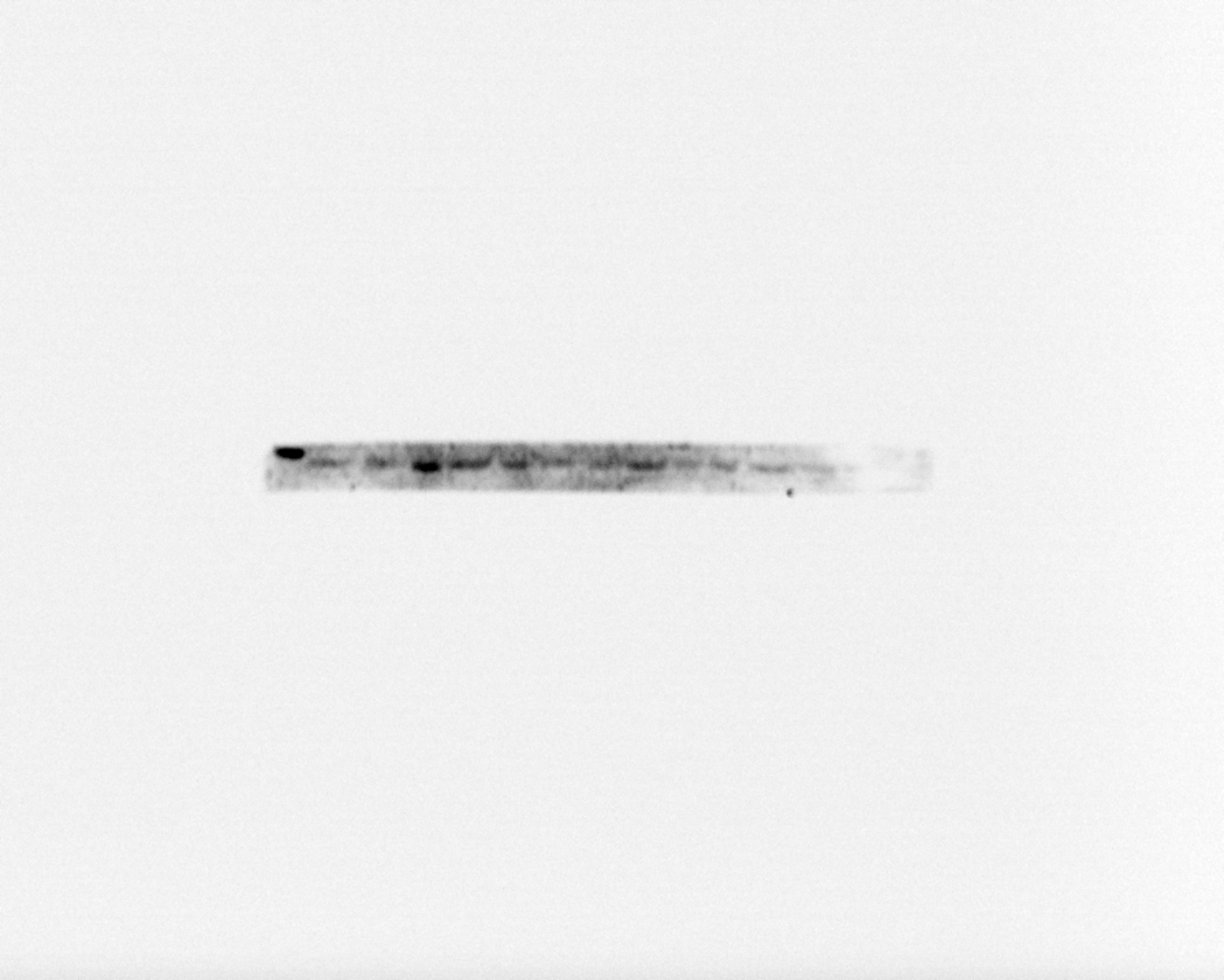

Supplement: Supplementary file 3 [file DataSheet2.ZIP › original blot images-Fig10/Fig10-p-ERK.tif]

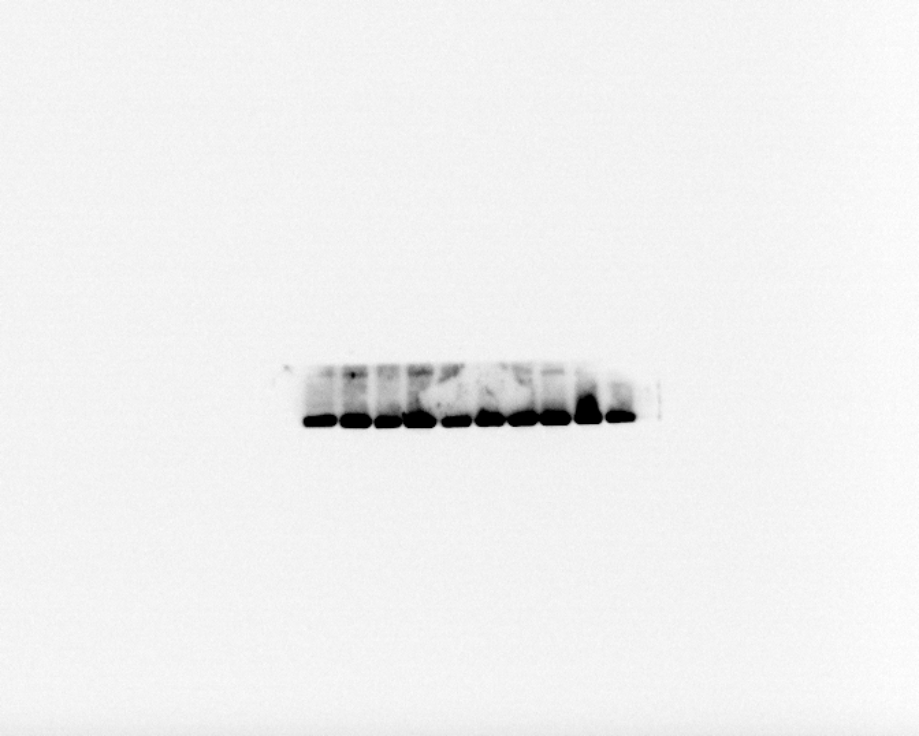

Supplement: Supplementary file 3 [file DataSheet2.ZIP › original blot images-Fig10/Fig10-AKT.tif]

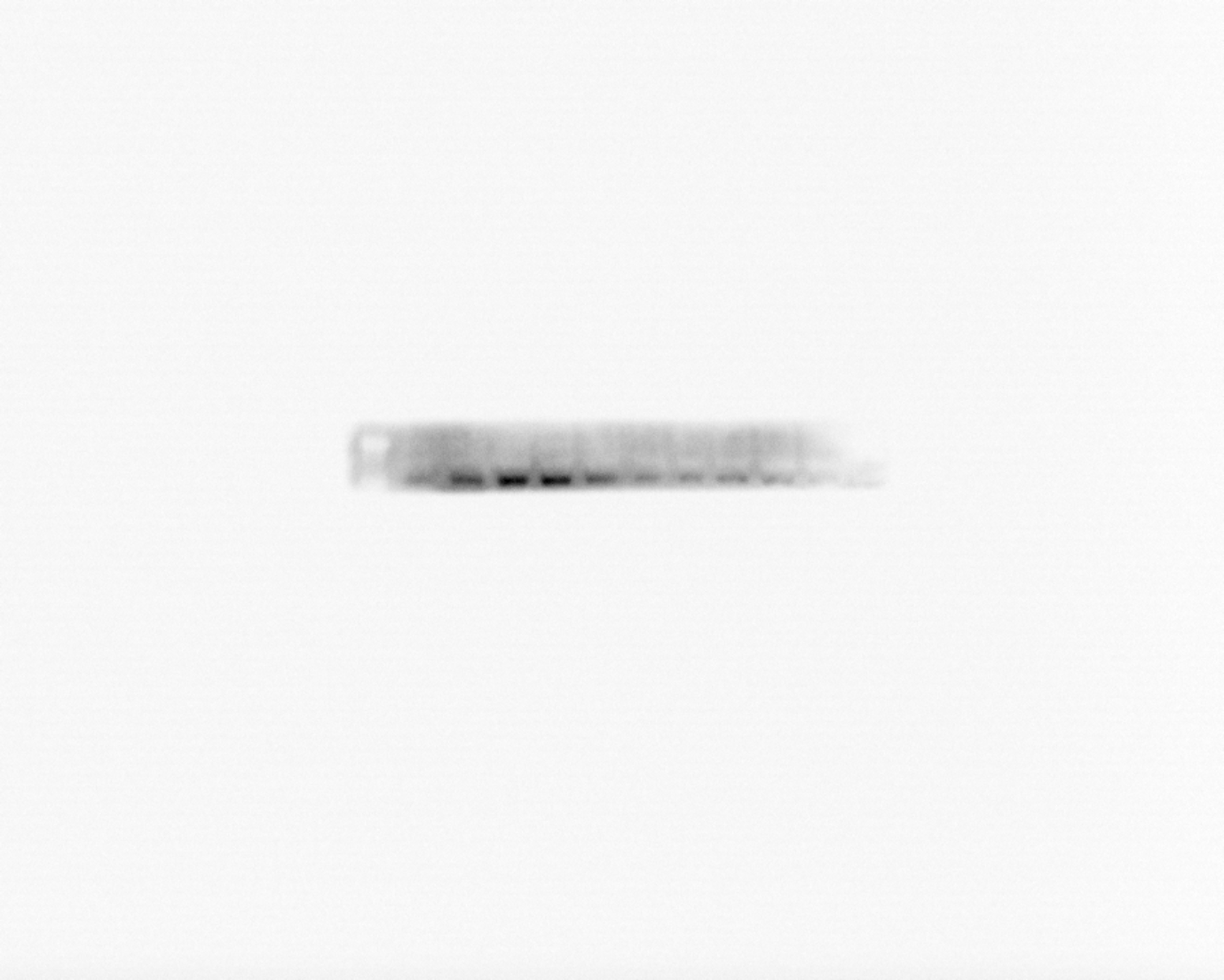

Supplement: Supplementary file 3 [file DataSheet2.ZIP › original blot images-Fig10/Fig10-p-AKT.tif]

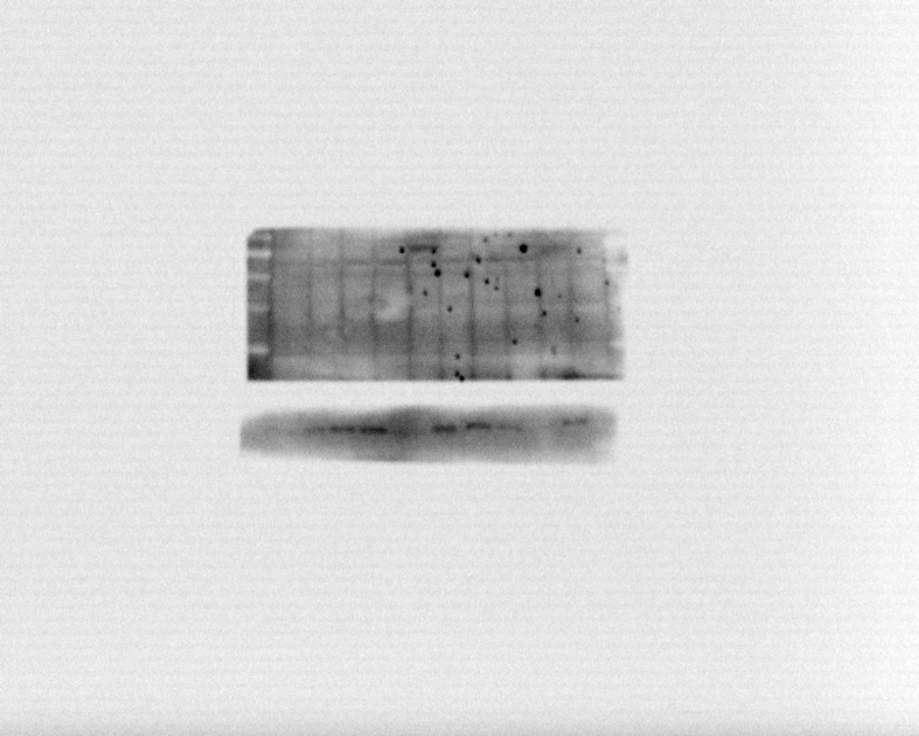

Supplement: Supplementary file 3 [file DataSheet2.ZIP › original blot images-Fig10/Fig10-p-smad3.tif]

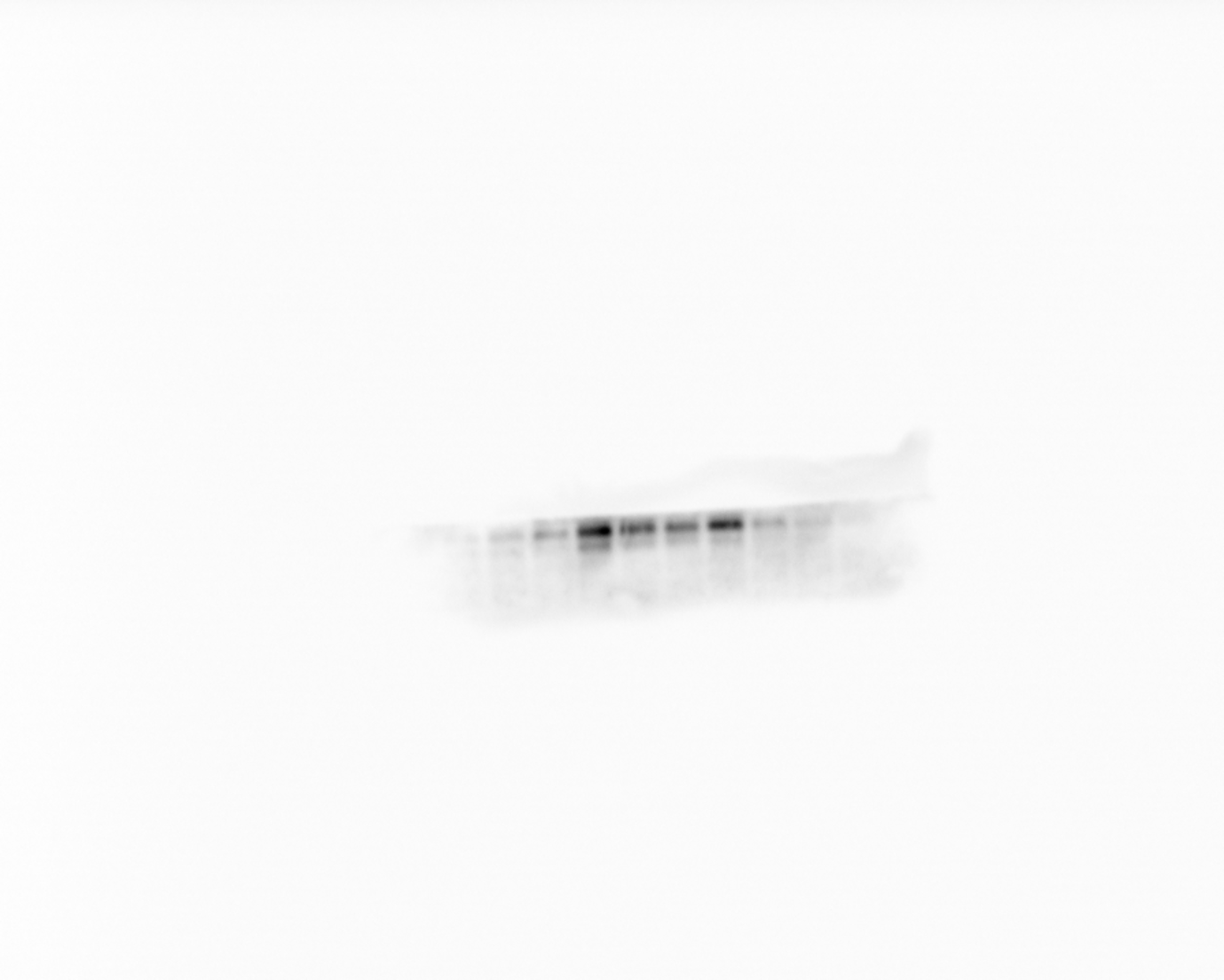

Supplement: Supplementary file 3 [file DataSheet2.ZIP › original blot images-Fig10/Fig10-P-Smad2.tif]
